# Supplementary material for: Metal-organic framework glasses with permanent accessible porosity
Source: Nat Commun. 2018 Nov 28;9:5042. doi: 10.1038/s41467-018-07532-z (PMC6262007; doi:10.1038/s41467-018-07532-z)
Supplement: Supplementary file 1 — Supplementary Information [file 41467_2018_7532_MOESM1_ESM.pdf]

Electronic Supplementary Information for

Metal-Organic Framework Glasses with Permanent Accessible Porosity

Chao Zhou et al,

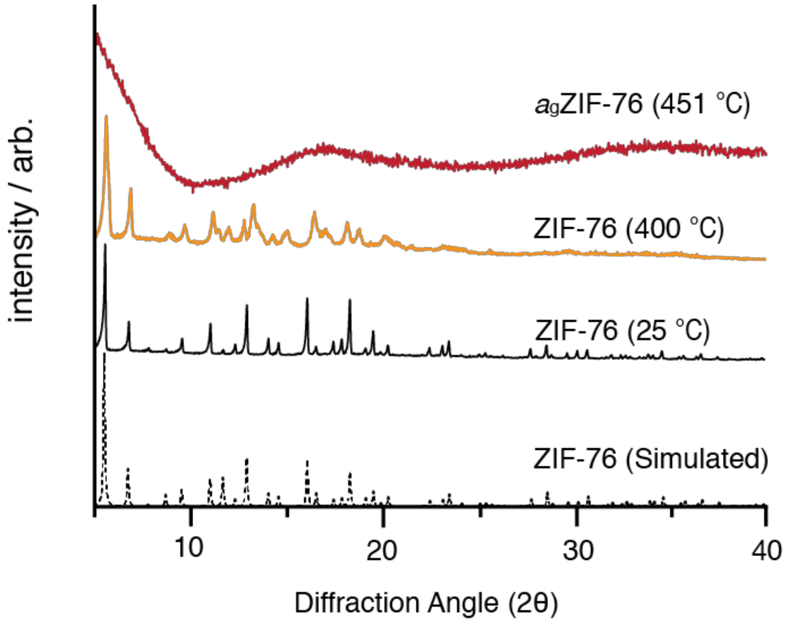

Supplementary Figure 1. X-ray powder diffraction patterns of ZIF-76. Experimental (as-synthesized), and those from samples quenched from 400 °C and 451 °C, are shown alongside the simulated pattern.

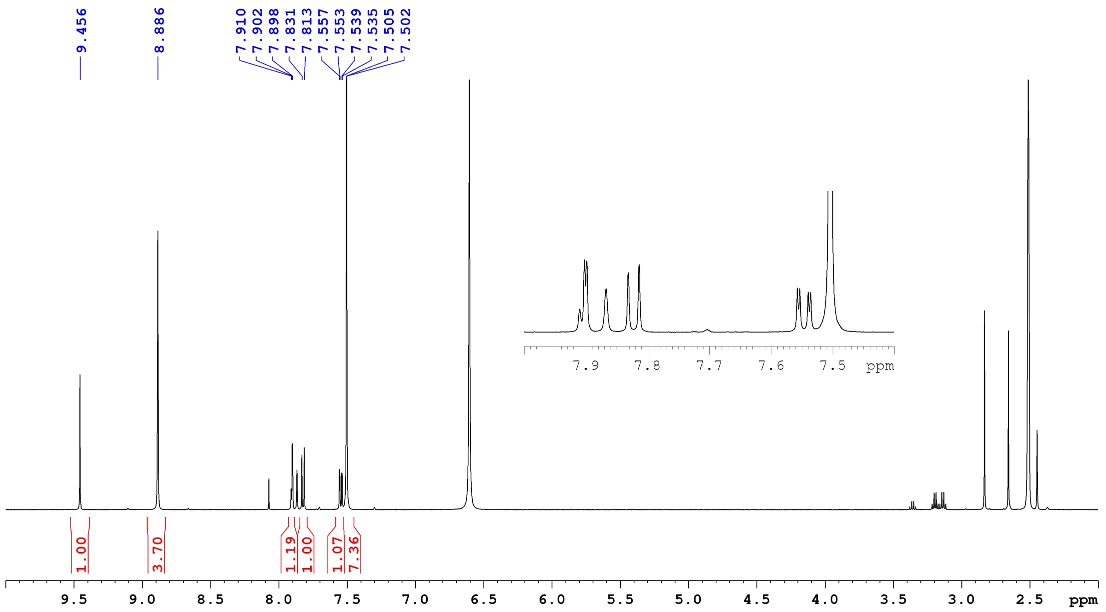

Supplementary Figure 2. ^{1}H NMR spectrum of as-synthesized ZIF-76 digested in DCl/DMSO-d$_6$. NMR peak assignments: $Zn(Im)_{1.62}(ClbIm)_{0.38}$. ^{1}H NMR (500 MHz, DMSO-*d*$_6$): 2.5 (DMSO-d$_6$), 7.50 (C***H***C***H***N$_{Im}$), 7.54 (CC***H***CH$_{ClbIm}$), 7.82 (CCHC***H***$_{ClbIm}$), 7.90 (ClCC***H***CC$_{ClbIm}$), 8.89 (NC***H***N$_{Im}$), 9.46 (NC***H***N$_{ClbIm}$).

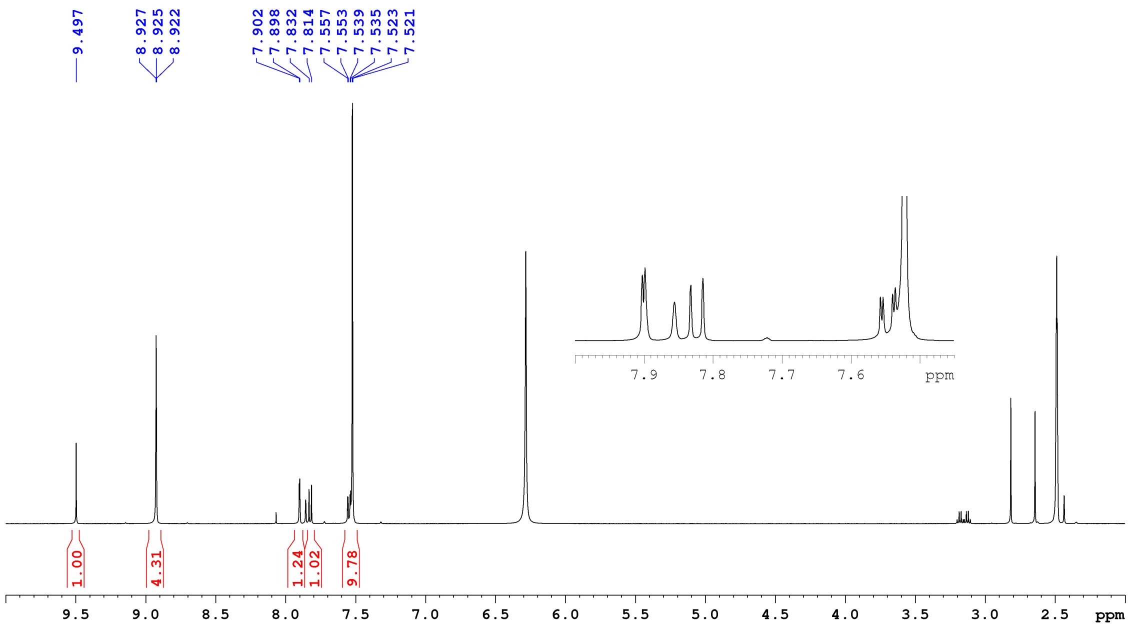

Supplementary Figure 3. ^{1}H NMR spectrum of a sample of ZIF-76 heated to 200 °C and cooled to room temperature, digested in DCl/DMSO-d$_6$. NMR peak assignments: Zn(Im)$_{1.62}$(ClbIm)$_{0.38}$. ^{1}H NMR (500 MHz, DMSO-*d*$_6$): 2.5 (DMSO-d$_6$), 7.52 (C***H***C***H***N$_{Im}$), 7.56 (CC***H***CH$_{ClbIm}$), 7.82 (CCHC***H***$_{ClbIm}$), 7.90 (ClCC***H***CC$_{ClbIm}$), 8.93 (NC***H***N$_{Im}$), 9.50 (NC***H***N$_{ClbIm}$).

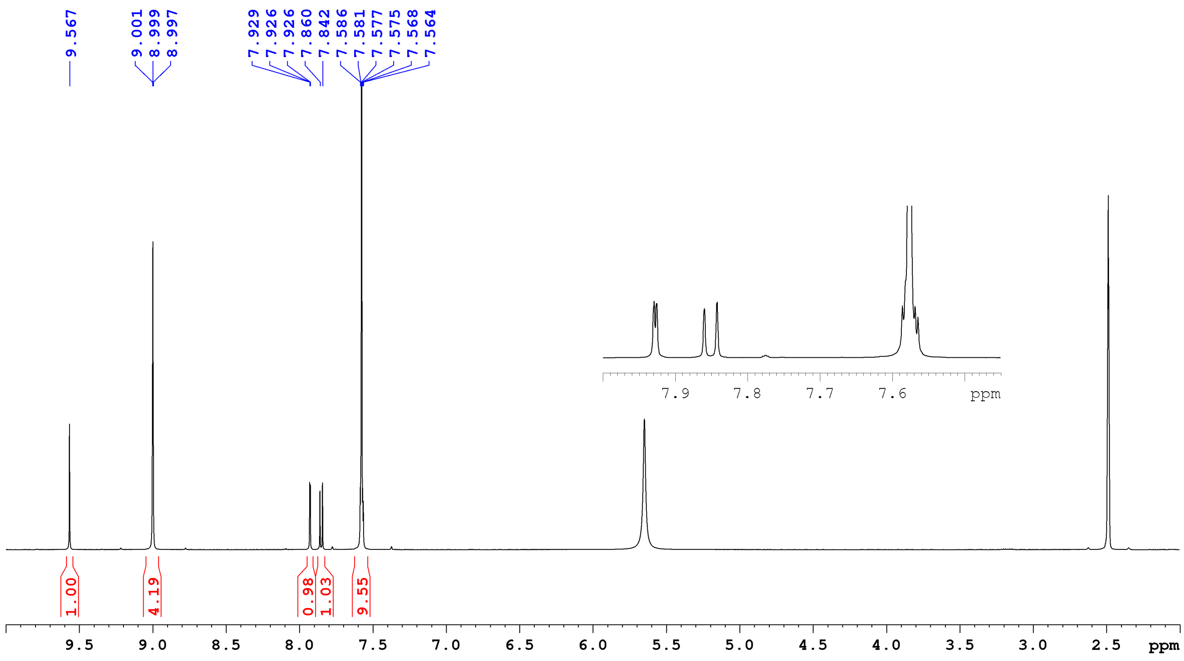

Supplementary Figure 4. ^{1}H NMR spectrum of a sample of ZIF-76 heated to 400 °C and cooled to room temperature, digested in DCl/DMSO-d$_6$. NMR peak assignments: Zn(Im)$_{1.62}$(ClbIm)$_{0.38}$. ^{1}H NMR (500 MHz, DMSO-*d*$_6$): 2.5 (DMSO-d$_6$), 7.56 (C***H***C***H***N$_{Im}$), 7.59 (CC***H***CH$_{ClbIm}$), 7.85 (CCHC***H***$_{ClbIm}$), 7.93 (ClCC***H***CC$_{ClbIm}$), 9.00 (NC***H***N$_{Im}$), 9.57 (NC***H***N$_{ClbIm}$).

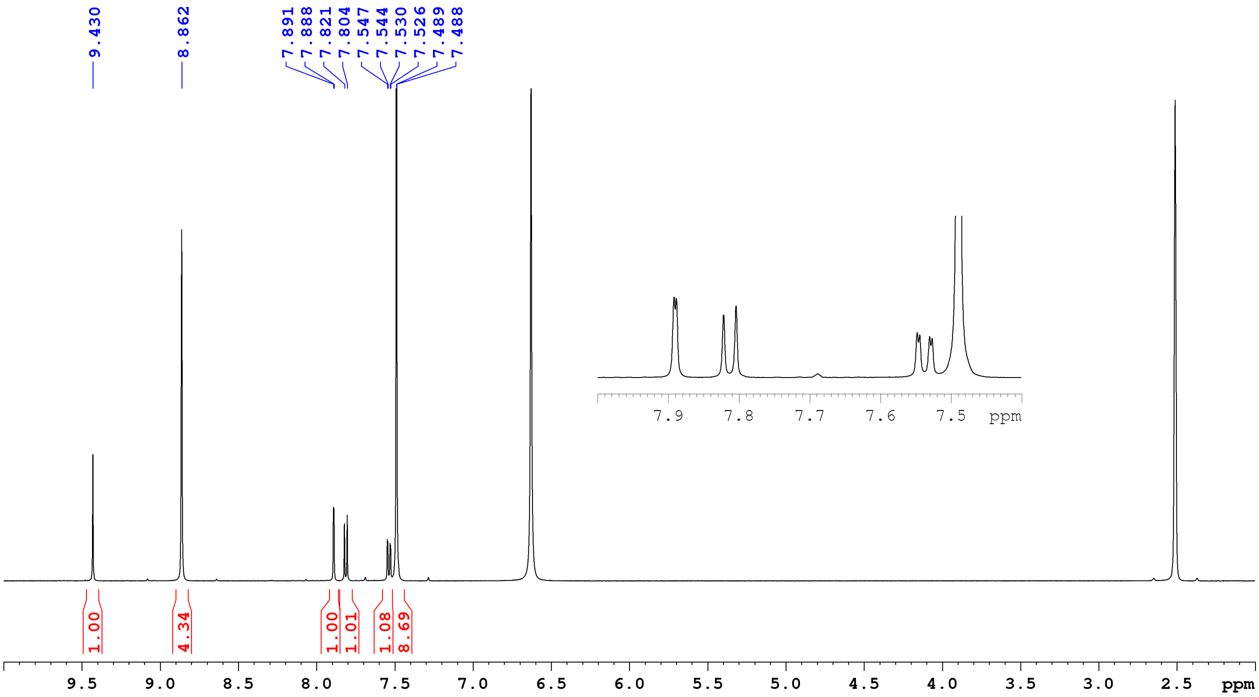

Supplementary Figure 5. ^{1}H NMR spectrum of a_gZIF-76 digested in DCl/DMSO-d$_6$. NMR peak assignments: Zn(Im)$_{1.62}$(ClbIm)$_{0.38}$. ^{1}H NMR (500 MHz, DMSO-d_6): 2.5 (DMSO-d$_6$), 7.49 (C***H***C***H***N$_{Im}$), 7.55 (CC***H***CH$_{ClbIm}$), 7.81 (CCHC***H***$_{ClbIm}$), 7.89 (ClCC***H***CC$_{ClbIm}$), 8.86 (NC***H***N$_{Im}$), 9.43 (NC***H***N$_{ClbIm}$).

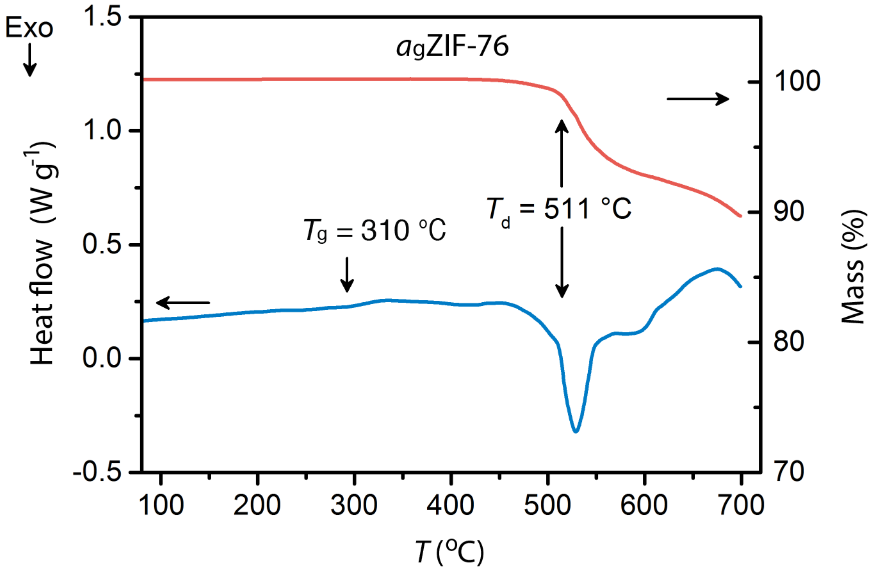

Supplementary Figure 6. Thermal analysis of a_gZIF-76. Isobaric heat capacity (C_p) and mass as a function of temperature (T) for a_gZIF-76. The DSC experiment was conducted in argon at a rate of 10 °C min^{-1}, to 700 °C.

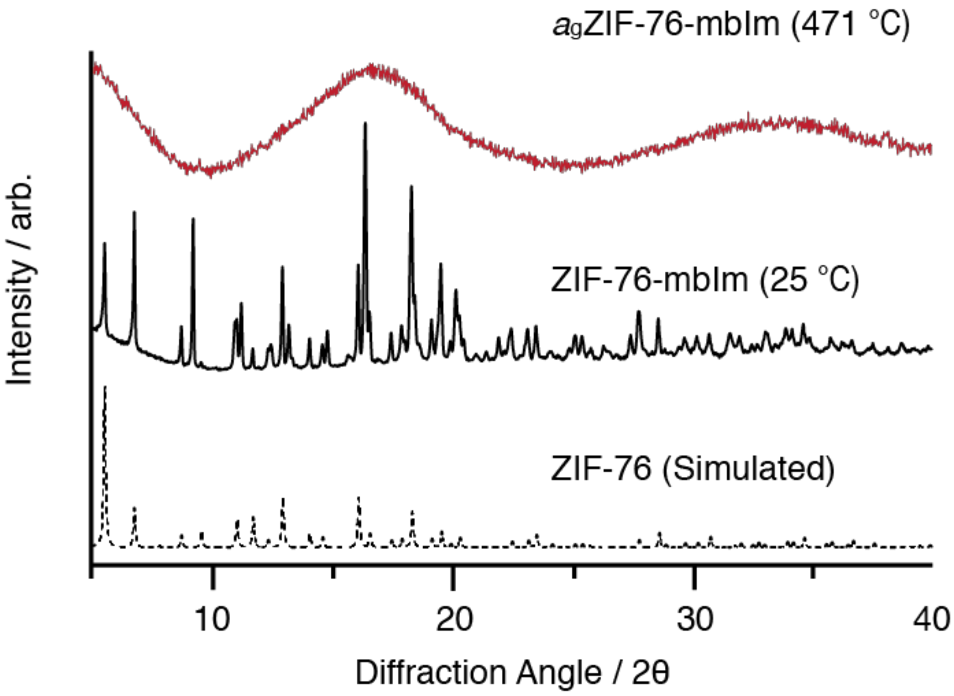

Supplementary Figure 7. X-ray powder diffraction patterns of ZIF-76-mbIm. Experimental (as-synthesized), and that from a sample quenched from 471 °C, are shown alongside the simulated pattern for ZIF-76.

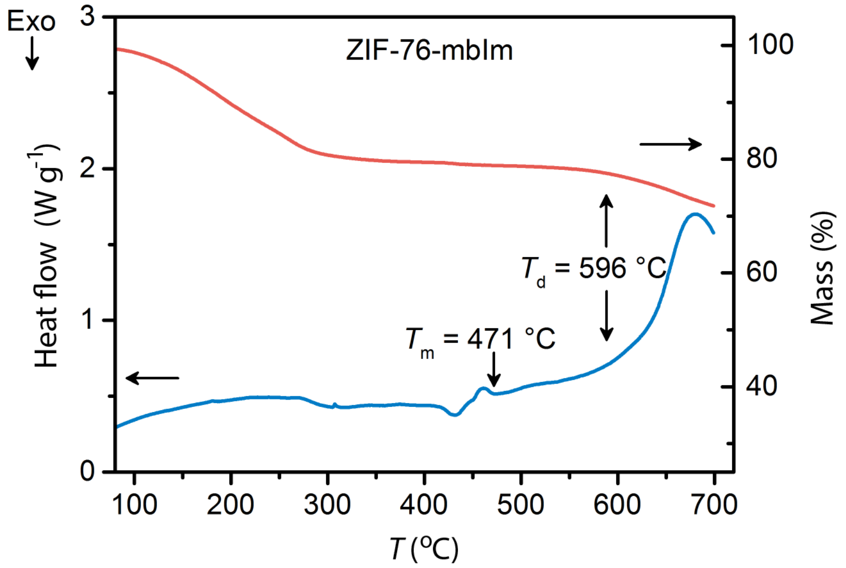

Supplementary Figure 8. Thermal analysis of ZIF-76-mbIm. Isobaric heat capacity (C_p) and mass as a function of temperature (T) for ZIF-76-mbIm. The DSC experiment was conducted in argon at a heating rate of 10 °C min^{-1}, to 700 °C.

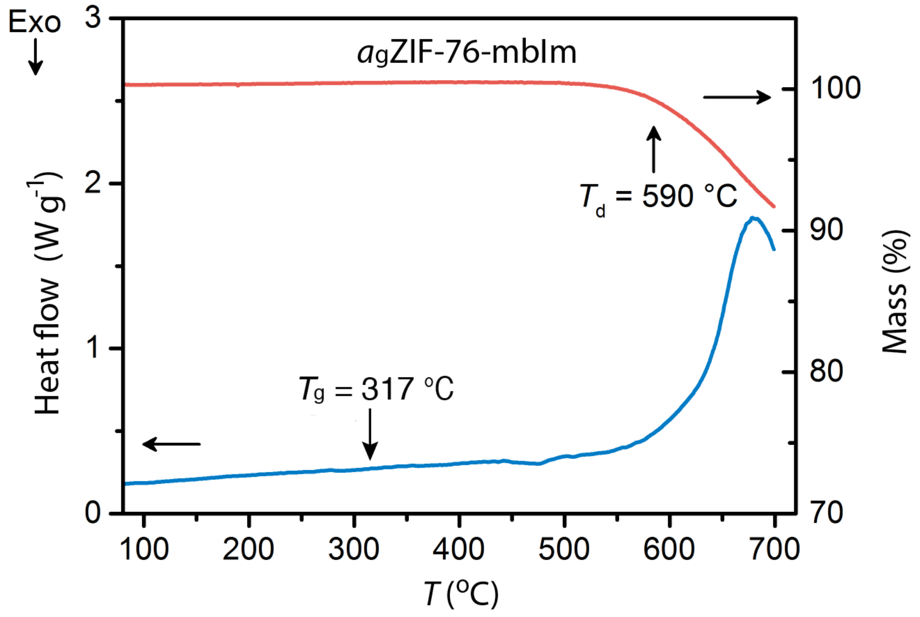

Supplementary Figure 9. Thermal analysis of a_gZIF-76-mbIm. Isobaric heat capacity (C_p) and mass as a function of temperature (T) for a_gZIF-76-mbIm. The DSC experiment was conducted in argon at a heating rate of 10 °C min^{-1}, to 700 °C.

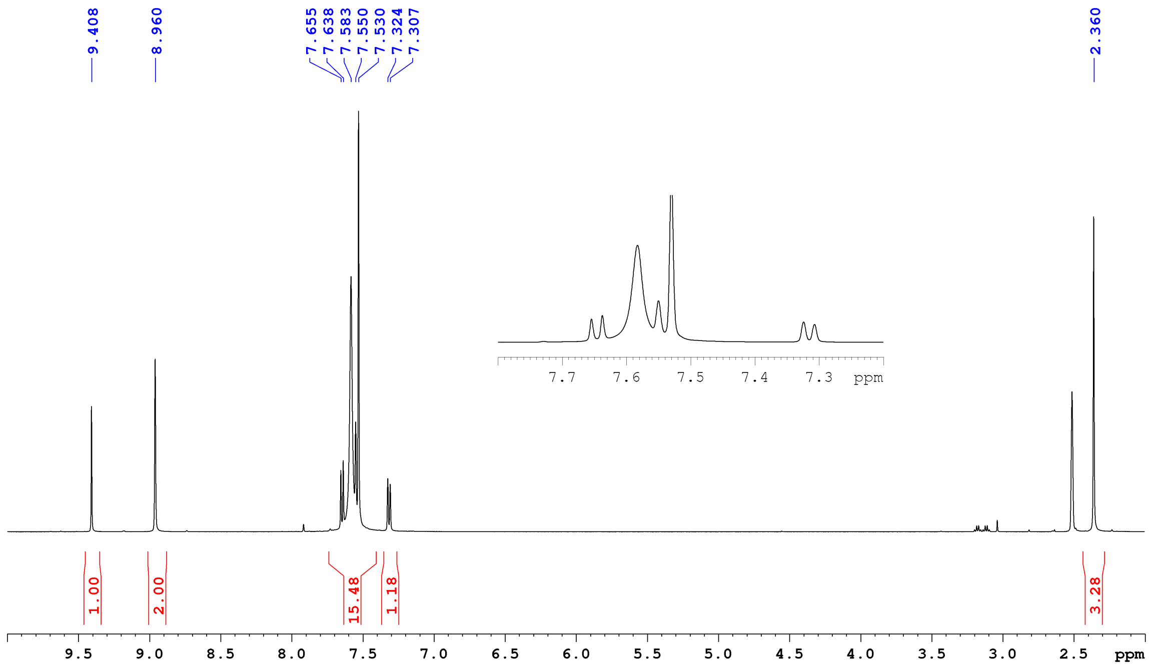

Supplementary Figure 10. ^{1}H NMR spectrum of ZIF-76-mbIm digested in DCl/DMSO-d$_6$. NMR peak assignments: ZIF-76-mbIm. (Zn(Im)$_{1.33}$(mbIm)$_{0.67}$). ^{1}H NMR (500 MHz, DMSO-*d*$_6$): 2.36 (C***H***$_{3\text{mbIm}}$), 2.5 (DMSO-d$_6$), 7.32 (CC***H***CH$_{\text{mbIm}}$), 7.54 (C***H***NC***H***$_{\text{Im}}$), 7.55 (CC***H***C(CH$_3$)$_{\text{mbIm}}$), 7.65 (CCHC***H***$_{\text{mbIm}}$), 8.96 (NC***H***N$_{\text{Im}}$), 9.41 (NC***H***N$_{\text{mbIm}}$).

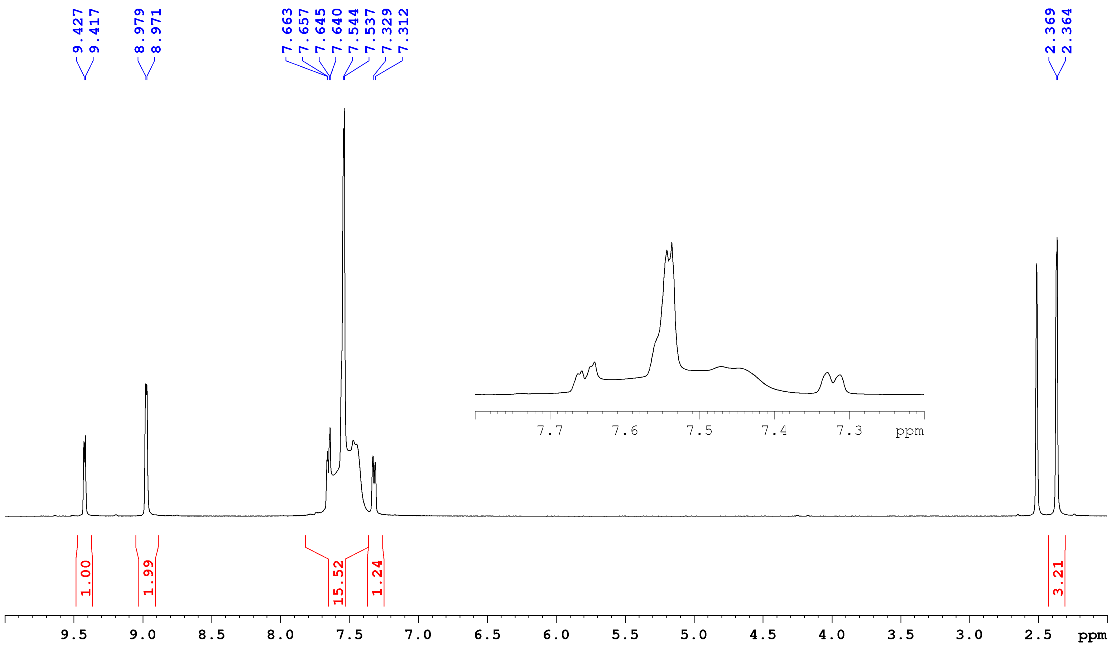

Supplementary Figure 11. ^{1}H NMR spectrum of a$_g$ZIF-76-mbIm digested in DCl/DMSO-d$_6$. NMR peak assignments: (Zn(Im)$_{1.33}$(mbIm)$_{0.67}$). ^{1}H NMR (500 MHz, DMSO-*d*$_6$): 2.36 (C***H***$_{3\text{mbIm}}$), 2.5 (DMSO-d$_6$), 7.32 (CC***H***CH$_{\text{mbIm}}$), 7.54 (C***H***NC***H***$_{\text{Im}}$), 7.55 (CC***H***C(CH$_3$)$_{\text{mbIm}}$), 7.66 (CCHC***H***$_{\text{mbIm}}$), 8.98 (NC***H***N$_{\text{Im}}$), 9.42 (NC***H***N$_{\text{mbIm}}$).

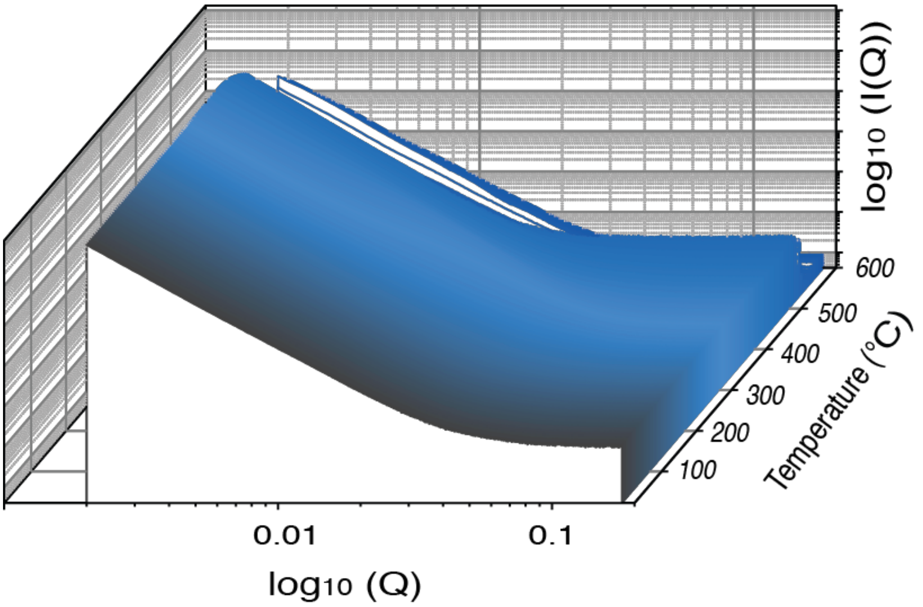

Supplementary Figure 12. Variable temperature resolved SAXS profile for ZIF-76. Heating range from 25 °C to 600 °C.

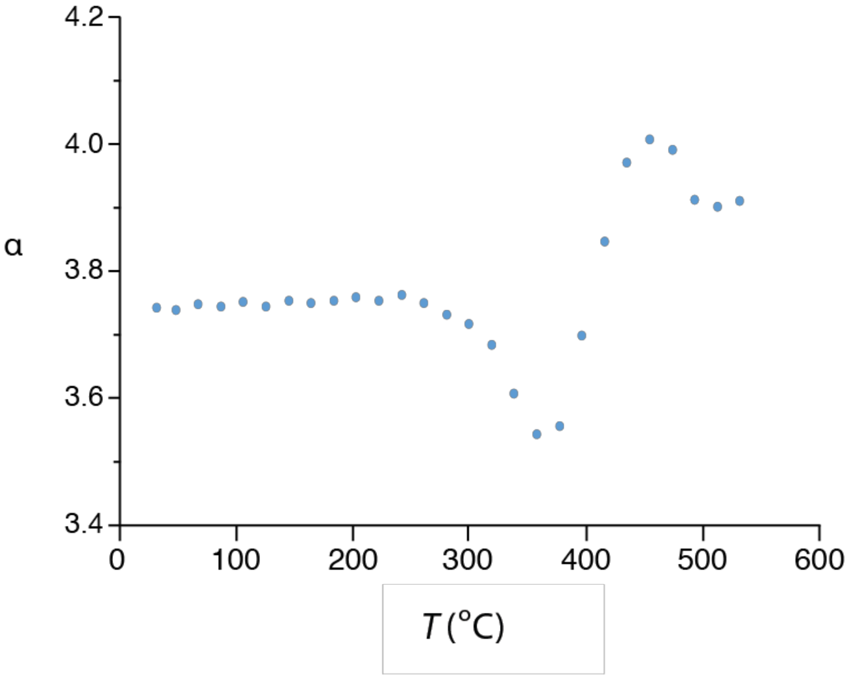

Supplementary Figure 13. Porod fitting of the variable temperature SAXS data. Experiments performed on a sample of crystalline, evacuated ZIF-76.

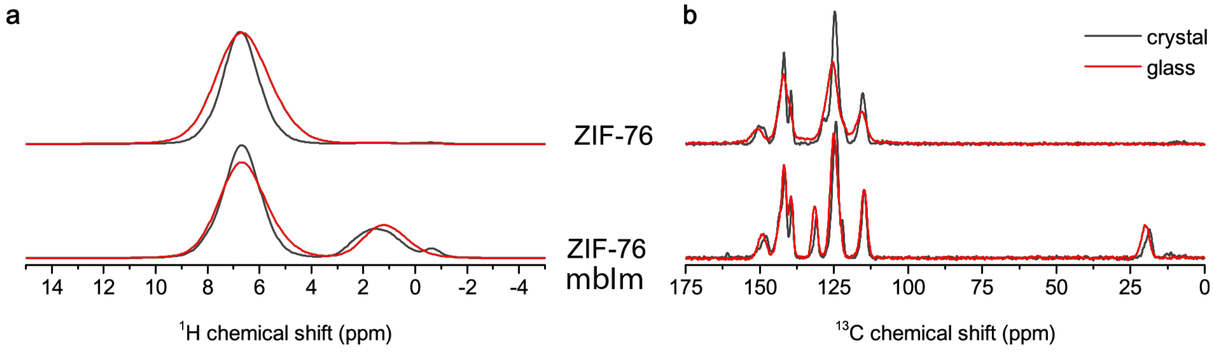

Supplementary Figure 14. Solid-State ^{1}H MAS (a) and (b) ^{13}C MAS NMR spectra.

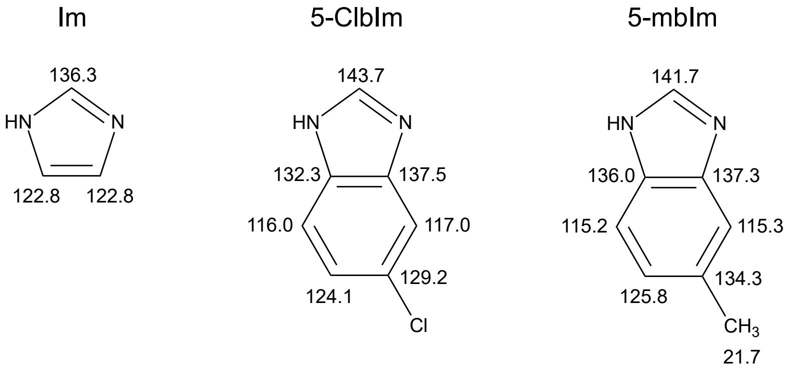

Supplementary Figure 15. ^{13}C isotropic chemical shifts of imidazole ligands. 5-chlorobenzimidazole (5-ClbImH) and 5-methylbenzimidazole (5-mbImH) and imidazole (ImH) species, as found in the literature. These chemical shifts enable tentative assignment of ^{13}C MAS NMR spectra.

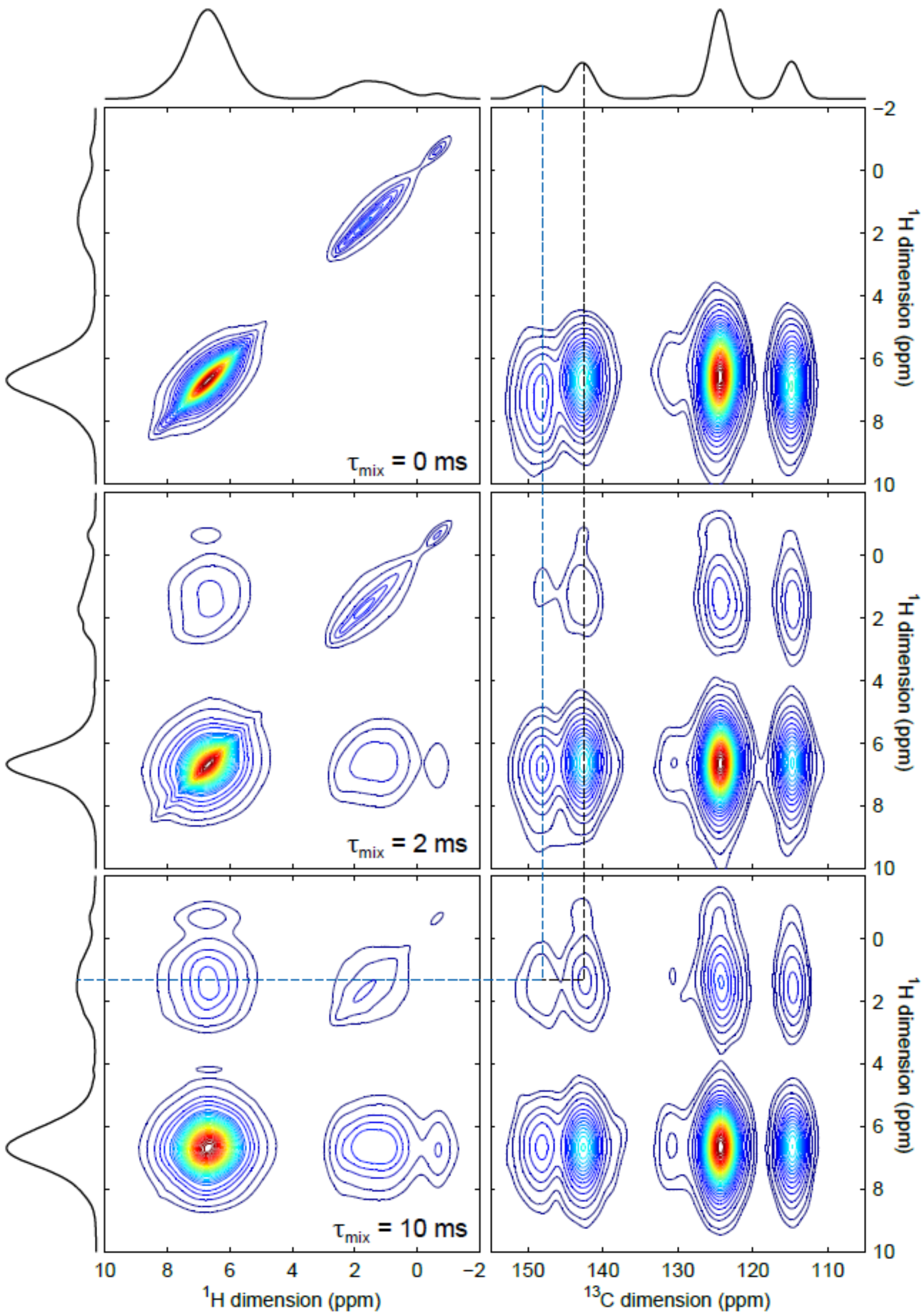

Supplementary Figure 16. Selected ^{1}H-detected and ^{13}C-detected 2D proton spin-diffusion NMR spectra of crystalline ZIF-76-mbIm. The absence of off-diagonal peaks at a mixing time of 0 ms indicates that proton polarization transfer has not occurred, whilst their steady appearance and strengthening in intensity from 2-10 ms is due to the polarization transfer taking place.

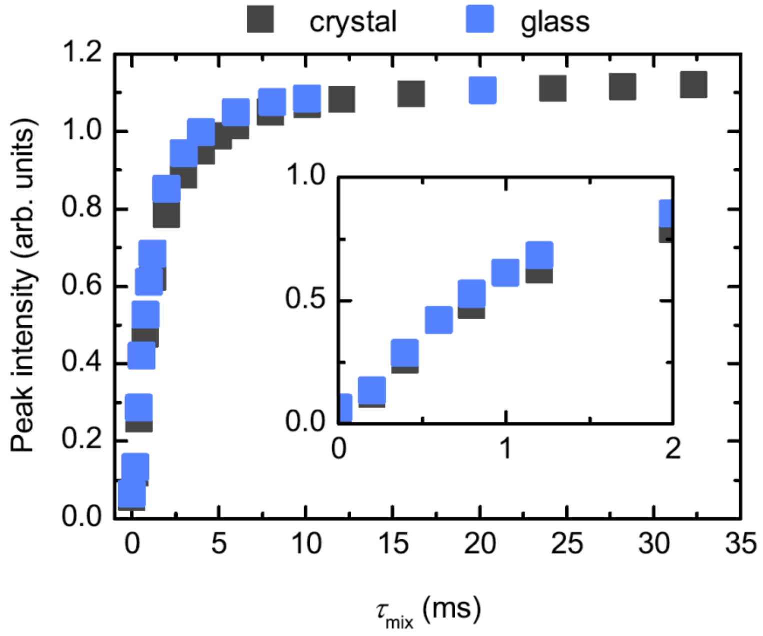

Supplementary Figure 17. Spin-diffusion curves obtained from the ^{1}H-detected measurements in crystalline ZIF-76-mbIm and a$_g$ZIF-76-mbIm.

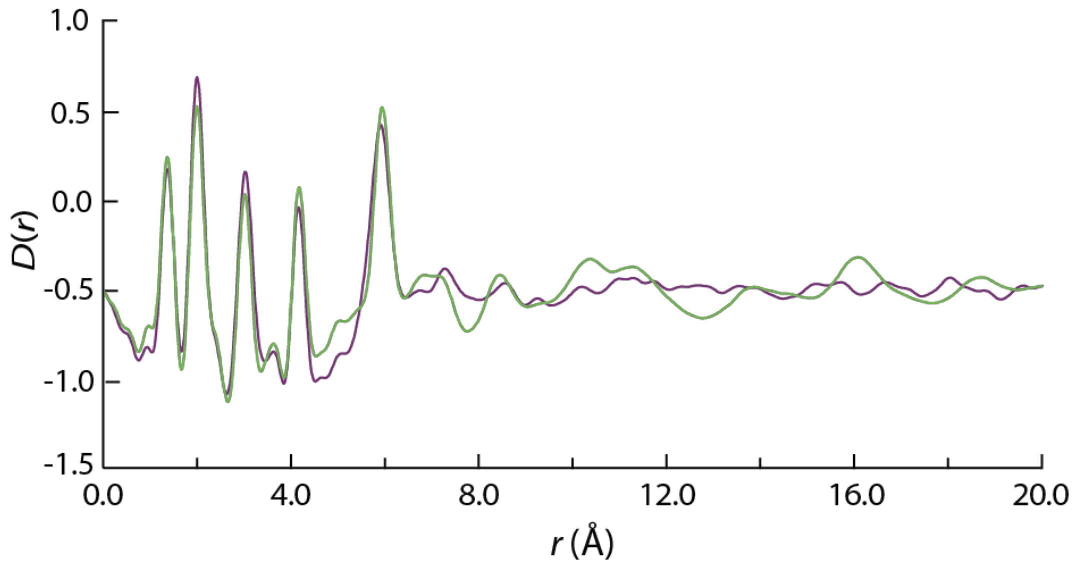

Supplementary Figure 18. Experimental X-ray total pair distribution functions D(*r*). ZIF-76-mbIm (green) and a$_g$ZIF-76-mbIm (purple).

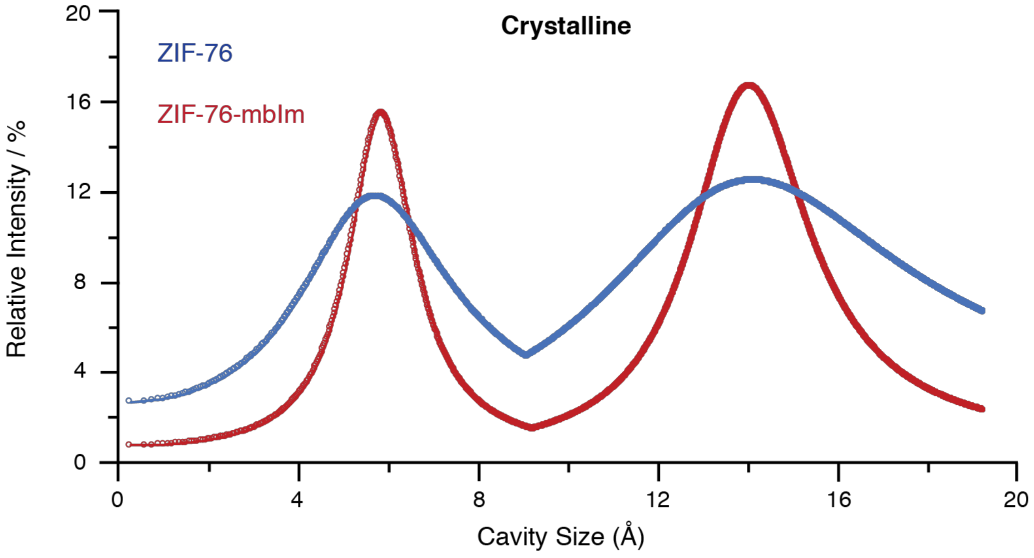

Supplementary Figure 19. PALS measurements on ZIF-76 and ZIF-76-mbIm. Results for the third and fourth components ($\tau 3$ and $\tau 4$) are represented as Gaussian distributions which correspond to the small and large cavities, respectively, and the Intensity is related to their relative number within the sample (I3 and I4).

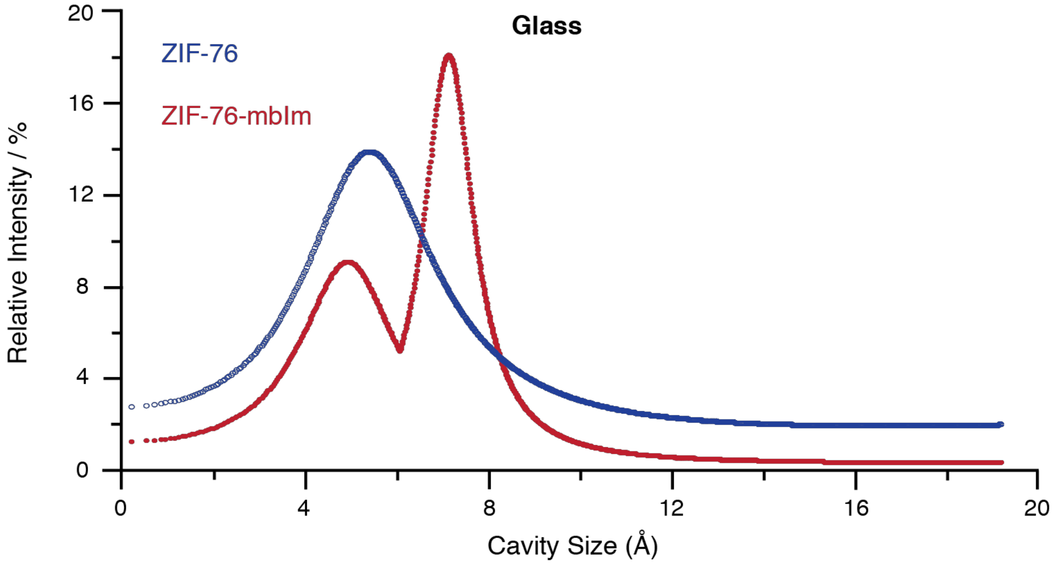

Supplementary Figure 20. PALS measurements on a_gZIF-76 and a_gZIF-76-mbIm. Results for the third and fourth components ($\tau 3$ and $\tau 4$) are represented as Gaussian distributions which correspond to the small and large cavities, respectively, and the Intensity is related to their relative number within the sample (I3 and I4).

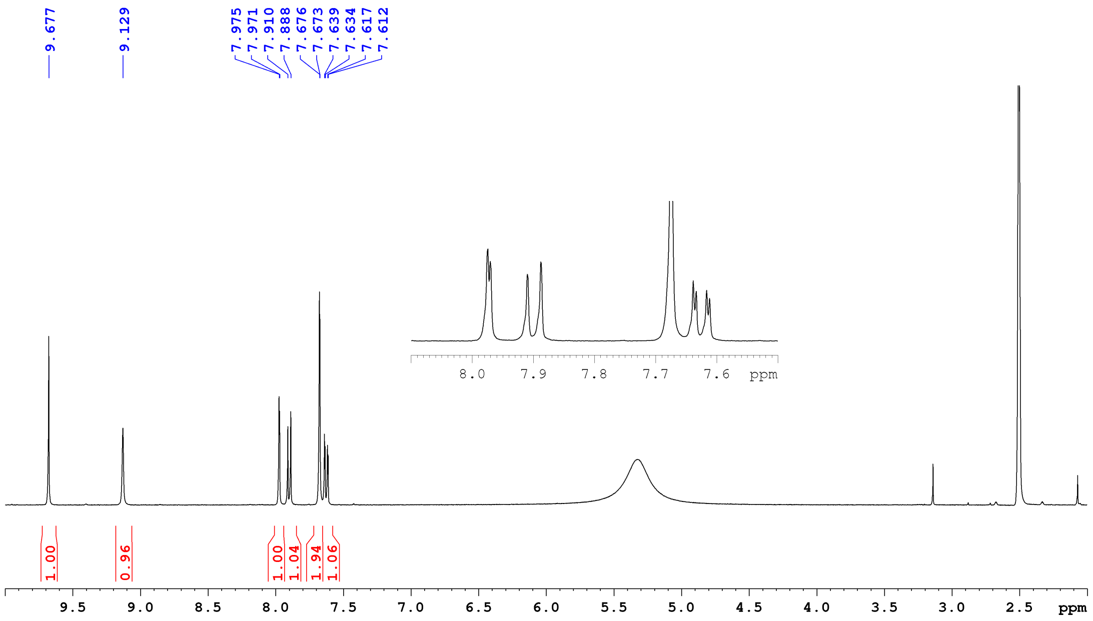

Supplementary Figure 21. ^{1}H NMR spectrum of ZIF-76, [Zn(Im)$_{1.0}$(5-ClbIm)$_{1.0}$], digested in DCl/DMSO-d$_6$. NMR peak assignments: ^{1}H NMR (500 MHz, DMSO-*d*$_6$): (Zn(Im)$_{1.0}$(5-ClbIm)$_{1.0}$). 2.5 (DMSO-d$_6$), 7.64 (CC***H***CH$_{\text{ClbIm}}$), 7.67 (C***H***C***H***N$_{\text{Im}}$), 7.90 (CCHC***H***$_{\text{ClbIm}}$), 7.97 (ClCC***H***CC$_{\text{ClbIm}}$), 9.13 (NC***H***N$_{\text{Im}}$), 9.68 (NC***H***N$_{\text{ClbIm}}$).

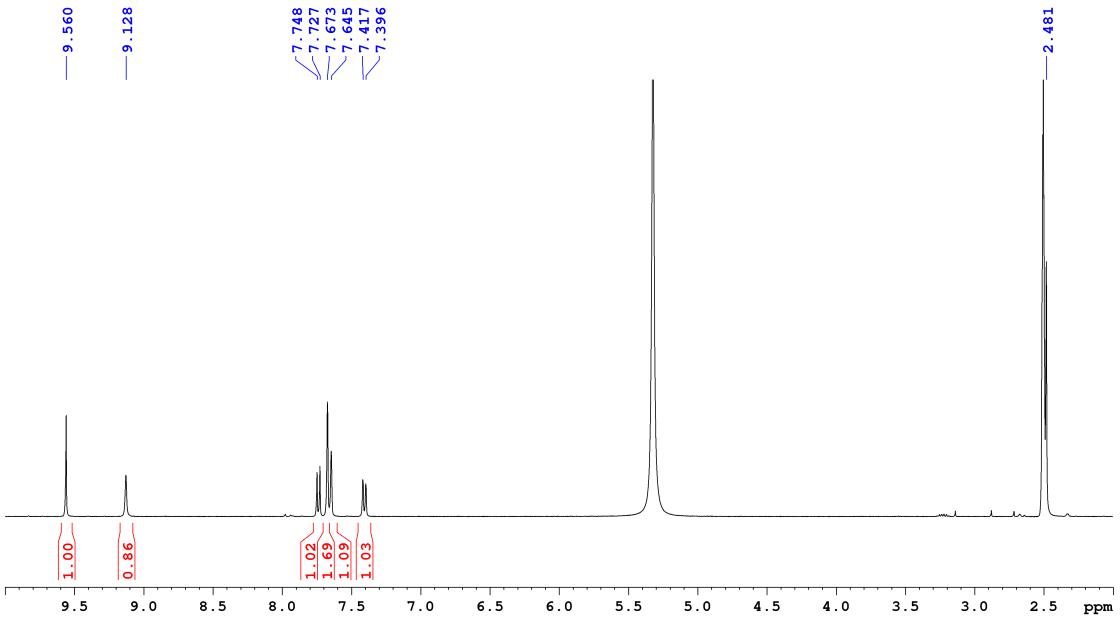

Supplementary Figure 22. ^{1}H NMR spectrum of ZIF-76-mbIm, [Zn(Im)$_{0.93}$(5-mbIm)$_{1.07}$], digested in DCl/DMSO-d$_6$. NMR peak assignments: (Zn(Im)$_{0.93}$(mbIm)$_{1.07}$). ^{1}H NMR (500 MHz, DMSO-*d*$_6$): 2.48 (C***H***$_{3\text{mbIm}}$), 2.5 (DMSO-d$_6$), 7.40 (CC***H***CH$_{\text{mbIm}}$), 7.65 (CC***H***C(CH$_3$)$_{\text{mbIm}}$), 7.67 (C***H***NC***H***$_{\text{Im}}$), 7.73 (CCHC***H***$_{\text{mbIm}}$), 9.13 (NC***H***N$_{\text{Im}}$), 9.56 (NC***H***N$_{\text{mbIm}}$).

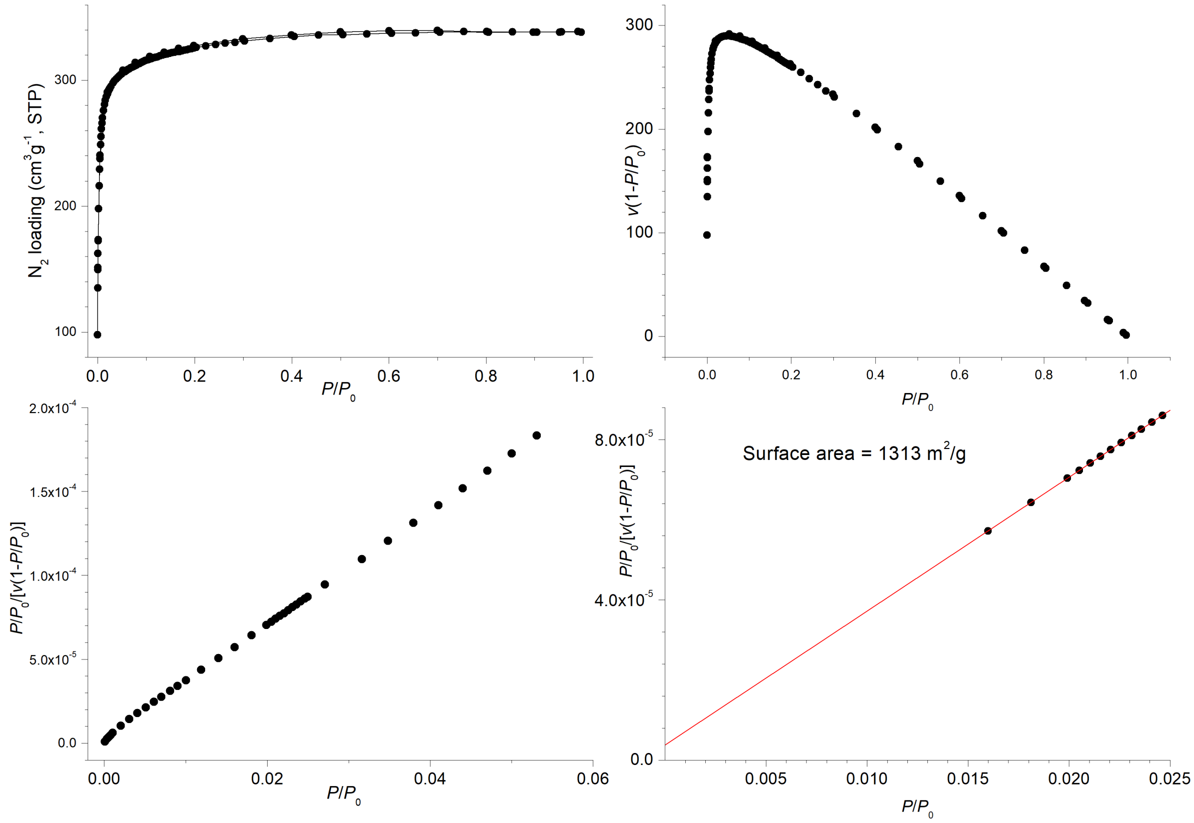

Supplementary Figure 23. N_2 adsorption isotherm at 77 K and BET surface area plots for ZIF-76, [Zn(Im)$_{1.0}$(5-ClbIm)$_{1.0}$].

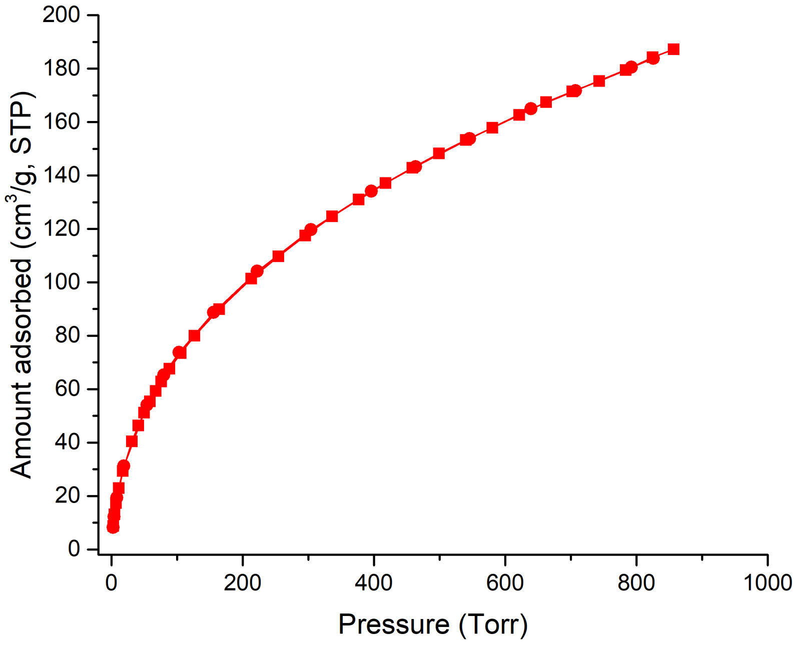

Supplementary Figure 24. H_2 adsorption isotherm at 77 K for ZIF-76, [Zn(Im)$_{1.0}$(5-ClbIm)$_{1.0}$] (circles = adsorption; squares = desorption).

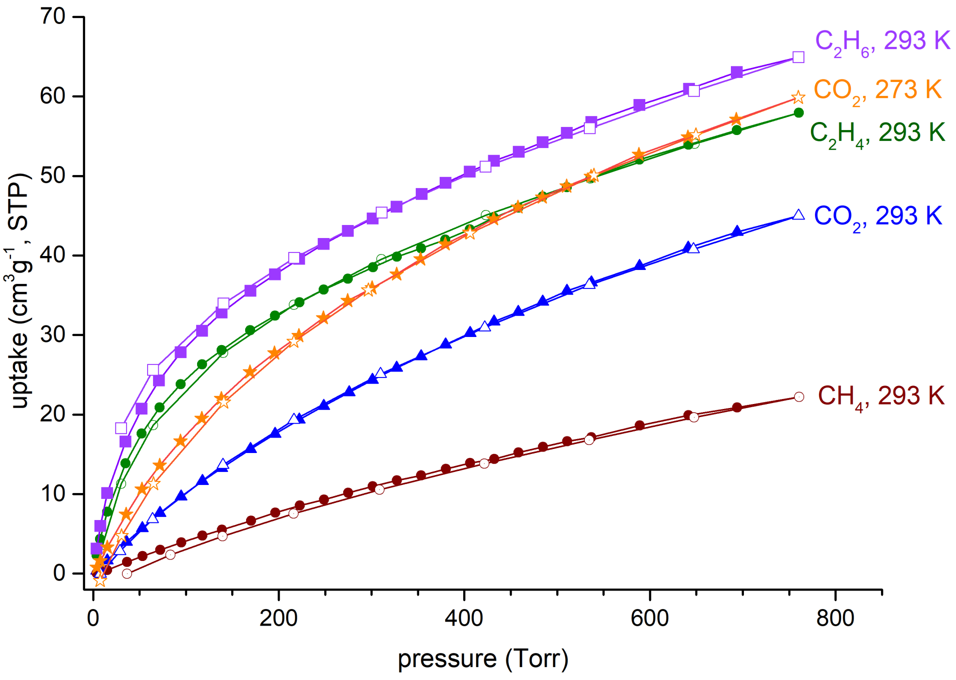

Supplementary Figure 25. Gas adsorption isotherms at 273K and 293 K for ZIF-76, [Zn(Im)$_{1.0}$(5-ClbIm)$_{1.0}$] (filled symbols = adsorption; empty = desorption).

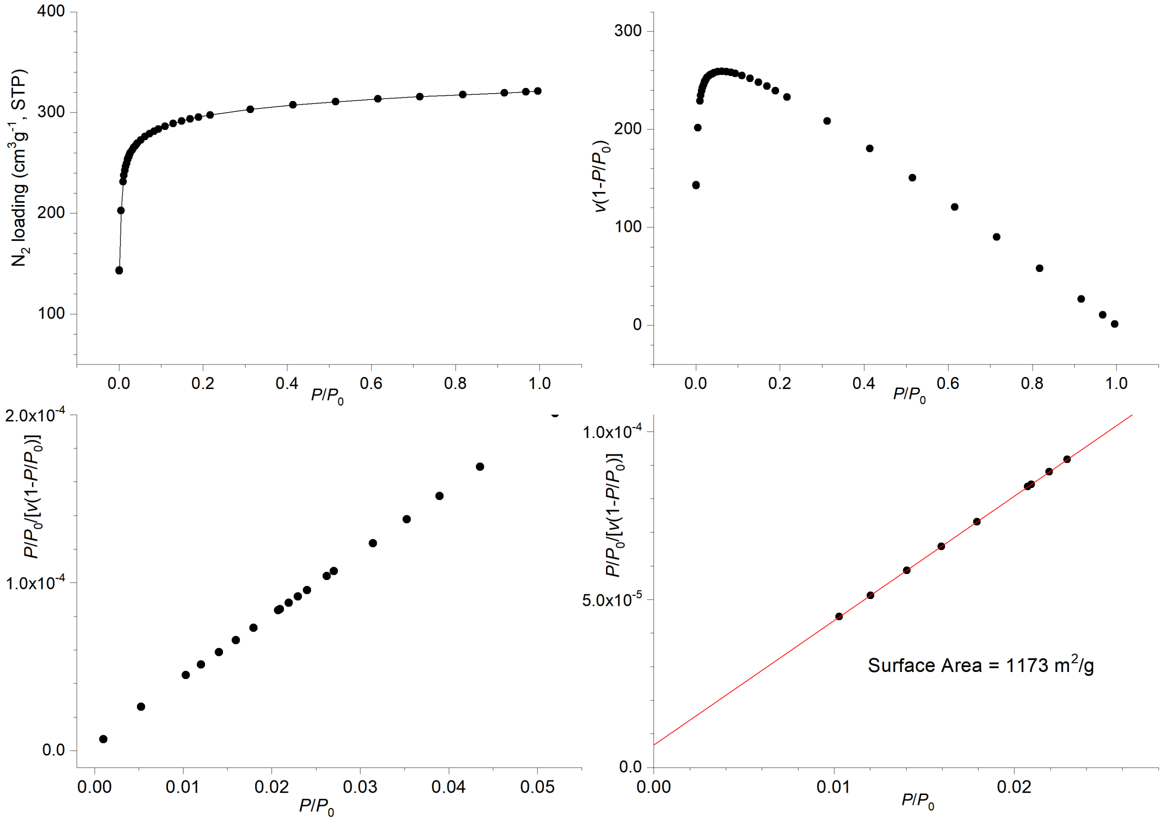

Supplementary Figure 26. N_2 adsorption isotherm at 77 K and BET surface area plots for ZIF-76-mbIm, [Zn(Im)$_{0.93}$(5-mbIm)$_{1.07}$].

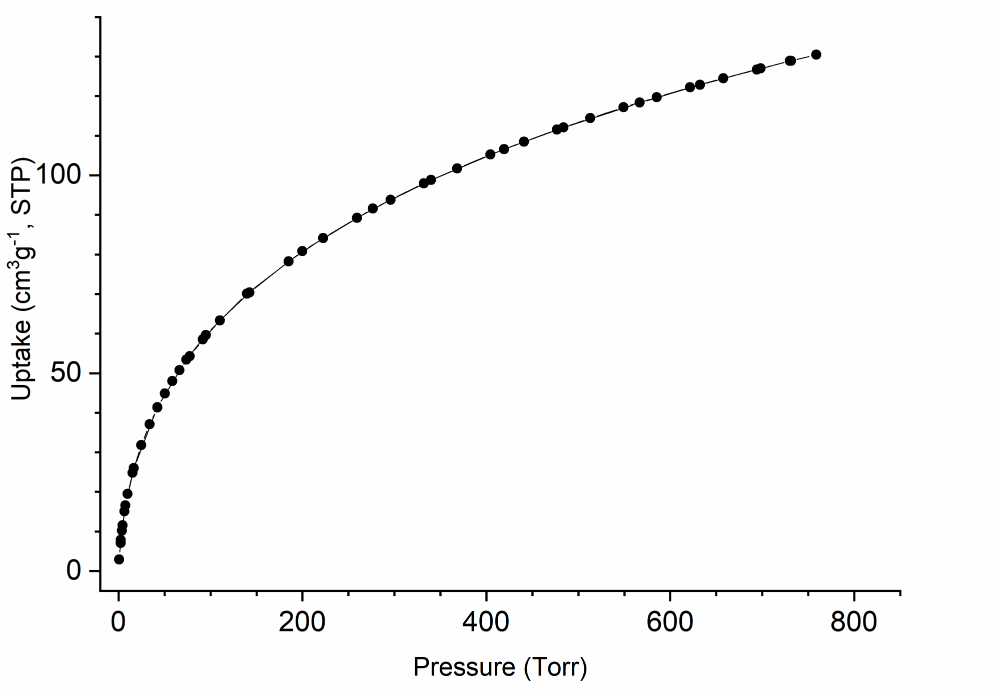

Supplementary Figure 27. H_2 adsorption isotherm at 77 K for ZIF-76-mbIm, [Zn(Im)$_{0.93}$(5-mbIm)$_{1.07}$].

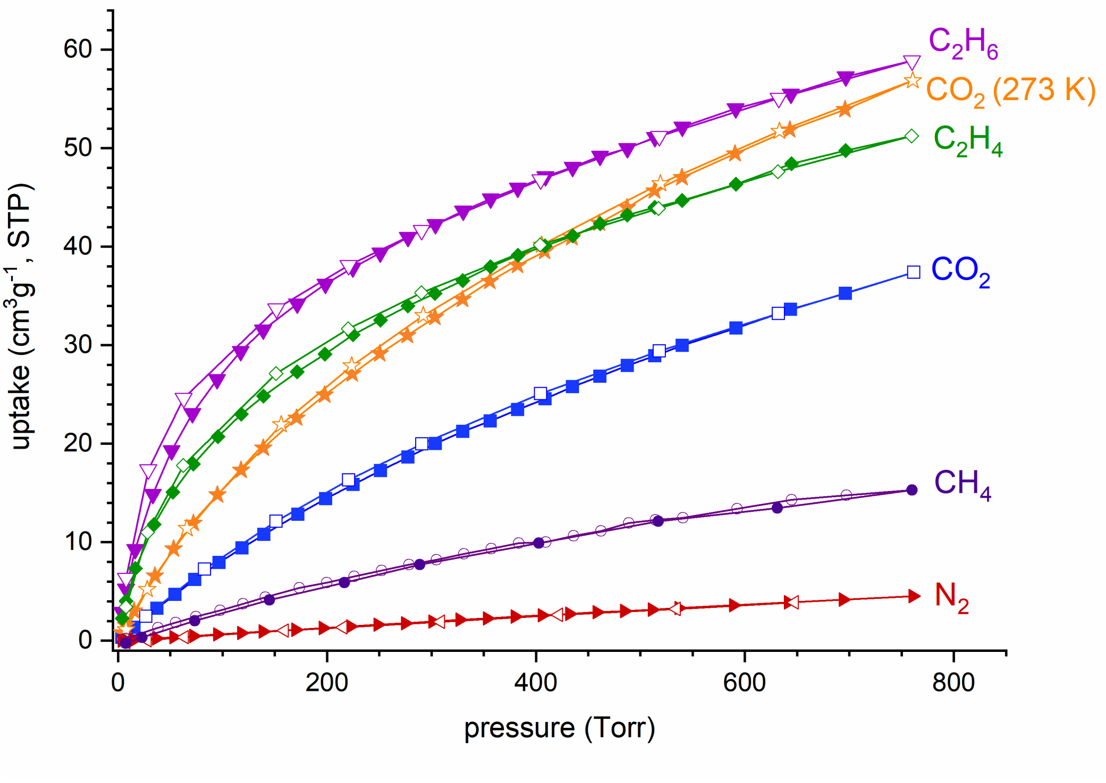

Supplementary Figure 28. Gas adsorption isotherms at 293 K (and 273 K where indicated) for ZIF-76-mbIm, [Zn(Im)$_{0.93}$(5-mbIm)$_{1.07}$] (filled symbols = adsorption; empty = desorption).

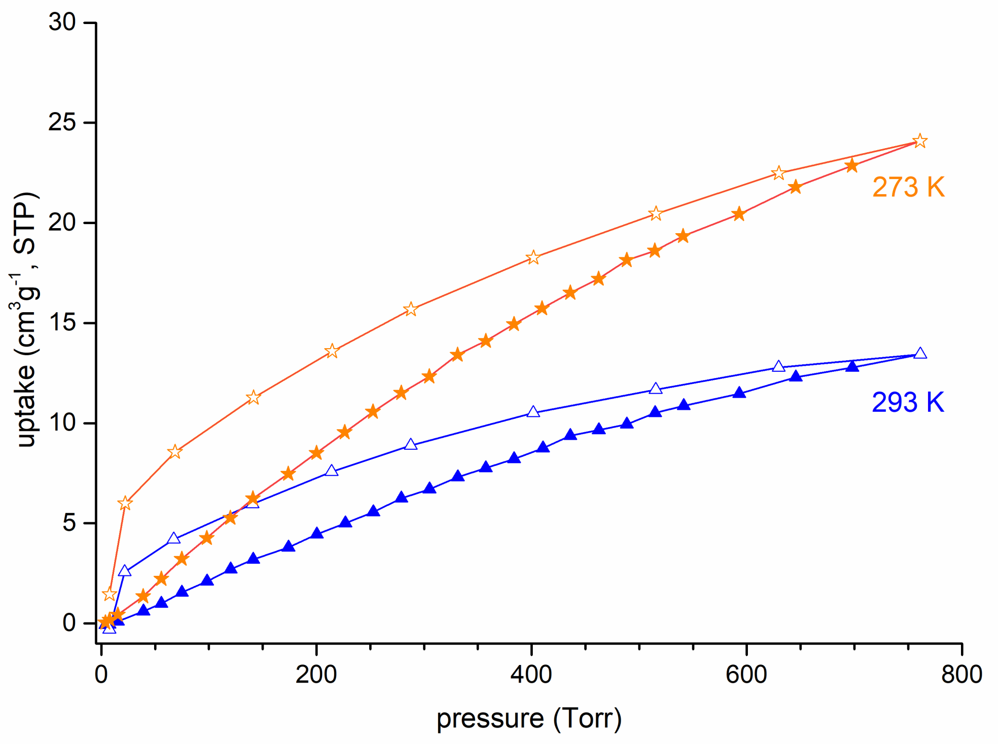

Supplementary Figure 29. CO_2 adsorption isotherms at 273K and 293 K for a$_g$ZIF-76, [Zn(Im)$_{1.0}$(5-ClbIm)$_{1.0}$] (filled symbols = adsorption; empty = desorption).

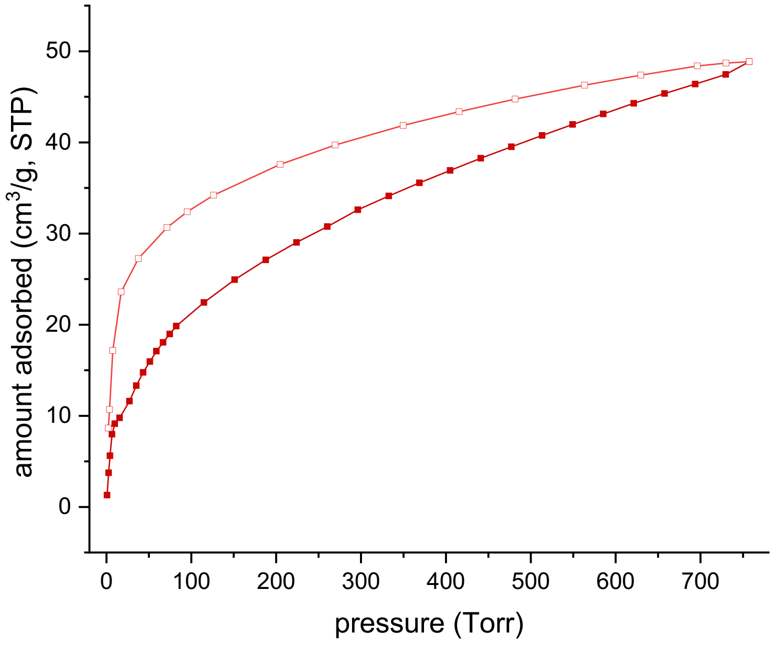

Supplementary Figure 30. H_2 adsorption isotherm for a$_g$ZIF-76-mbIm, [Zn(Im)$_{0.93}$(5-mbIm)$_{1.07}$], at 77 K (filled symbols = adsorption; empty = desorption).

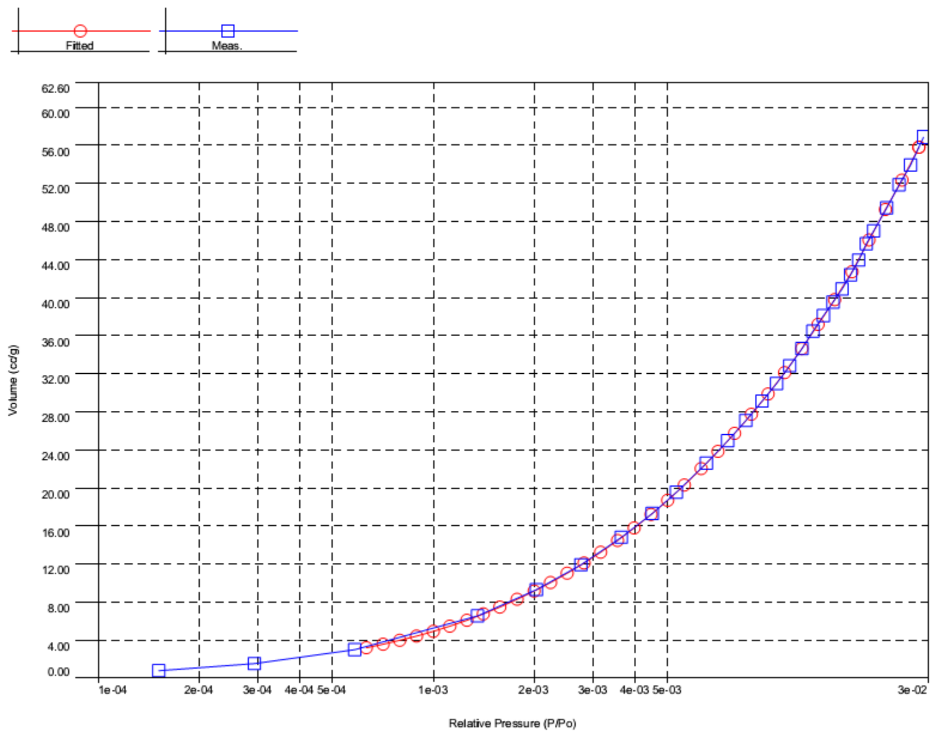

Supplementary Figure 31. NLDFT fitting of the CO_2 adsorption isotherm at 273 K of ZIF-76-mbIm, $[Zn(Im)_{0.93}(5\text{-}mbIm)_{1.07}]$.

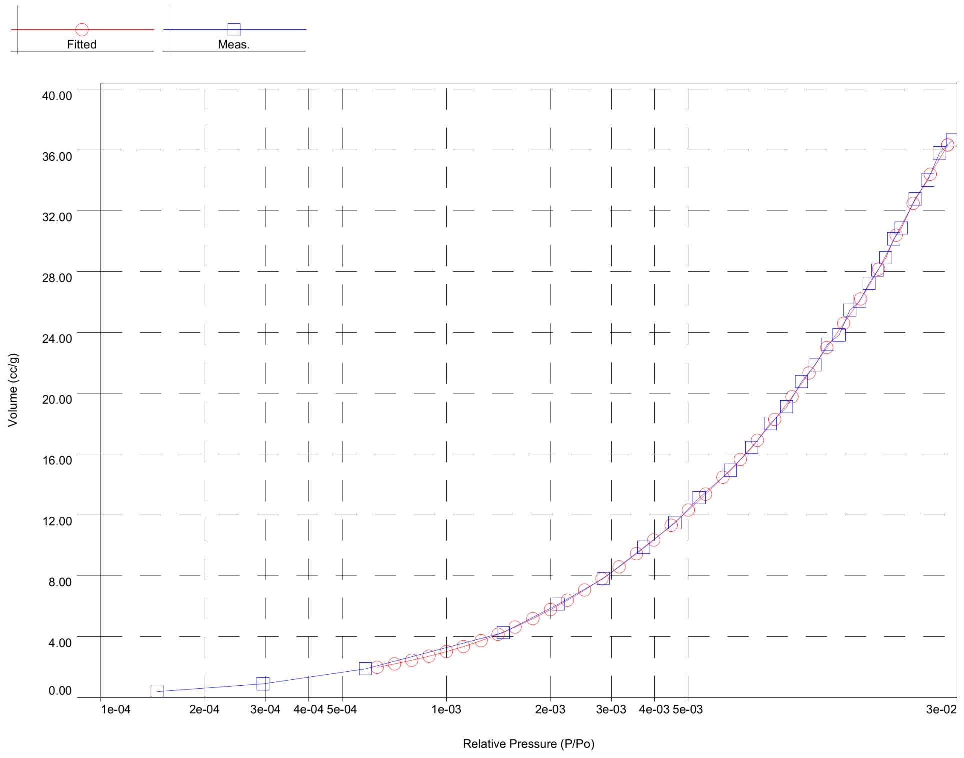

Supplementary Figure 32. NLDFT fitting of the CO_2 adsorption isotherm at 273 K of a_gZIF-76-mbIm, $[Zn(Im)_{0.93}(5\text{-}mbIm)_{1.07}]$.

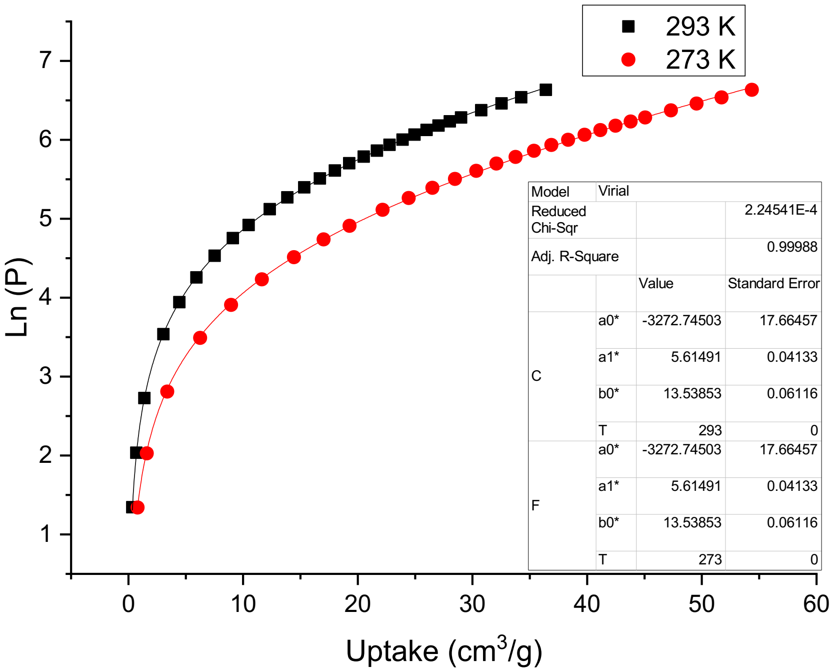

Supplementary Figure 33. Virial fitting of CO_2 adsorption isotherms at 273 K and 293 K for ZIF-76-mbIm, [Zn(Im)$_{0.93}$(5-mbIm)$_{1.07}$], used to calculate the isosteric heat of adsorption (Q_{st}).

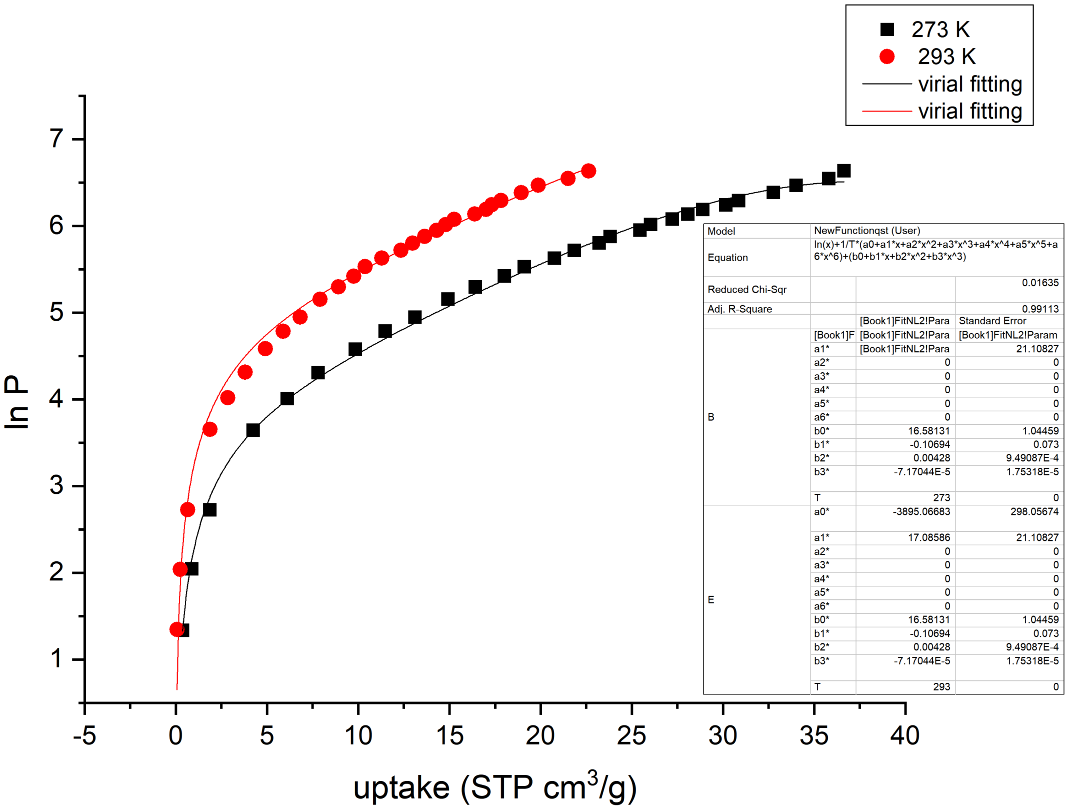

Supplementary Figure 34. Virial fitting of the CO_2 adsorption isotherms at 273 K and 298 K for a$_g$ZIF-76-mbIm, [Zn(Im)$_{0.93}$(5-mbIm)$_{1.07}$], used to calculate the isosteric heat of adsorption (Q_{st}).

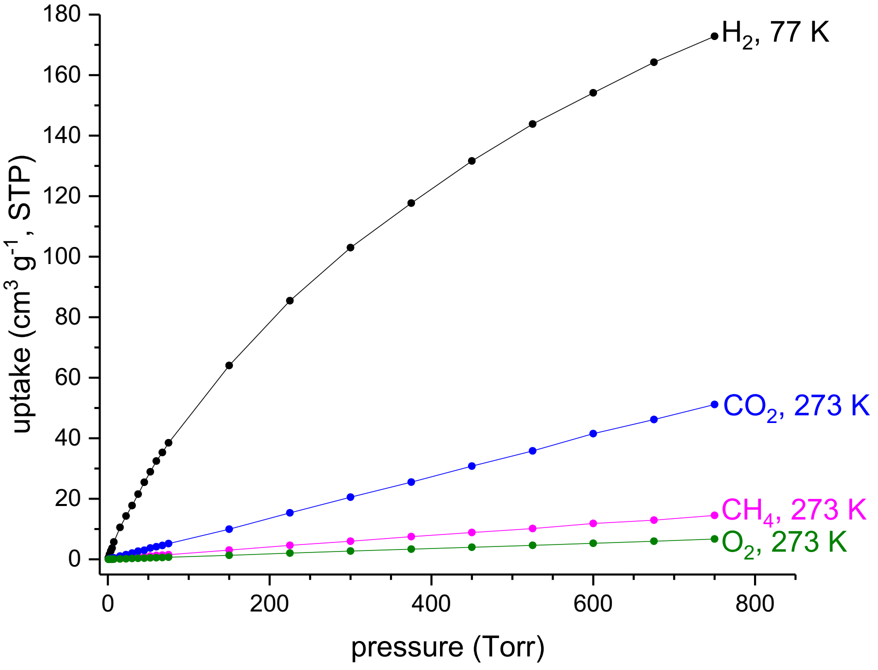

Supplementary Figure 35. Simulated H_2, CO_2, CH_4 and O_2 isotherms for ZIF-76.

Supplementary Tables

Supplementary Table 1. Positron annihilation lifetime spectroscopy data.

	Intensity		Lifetime	
Sample	I3 (%)	I4 (%)	τ3 (ns)	τ4 (ns)
ZIF-76	5.8 ±0.2	6.2 ± 0.1	1.999 ± 0.05	11.934 ± 0.20
a_gZIF-76	6.3 ± 0.1		1.826 ± 0.02	
ZIF-76-mbIm	15.4 ± 0.3	16.6 ± 0.1	2.068 ± 0.03	11.741 ± 0.07
a_gZIF-76-mbIm	9.0 ± 1.6	18.0 ± 1.8	1.554 ± 0.14	2.913 ± 0.08

Supplementary Table 2. Gas adsorption properties for crystalline ZIF-76 and ZIF-76-mbIm. The data are given as volumetric uptake (mL STP / g) at a pressure of 1 bar.

Gas	H_2	CO_2	Ar	O_2	N_2	CH_4
Temperature / K	77	273	77	273	77	273
ZIF-76 [a]						
Experimental	187	59.9	-	-	338	25.2
Simulated	172.8	51.2	321.4	6.7	275	14.6
ZIF-76-mbIm						
Experimental	130.5	54.4	-	27.4	339	44.1

[a] Calculations performed on $[Zn(Im)_{1.5}(5\text{-ClbIm})_{0.5}]$ while experimental isotherms were measured on $[Zn(Im)_{1.0}(5\text{-ClbIm})_{1.0}]$.

Supplementary Table 3. Interaction potential parameters used for gas molecules.

Molecule	Atom	σ (Å)	ε/k_B (K)	q(e)
H_2	H_2	2.96	34.20	-
CH_4	CH_4	3.73	148.00	-
Ar	Ar	3.410	119.50	-
CO_2	C	2.80	27.02	0.700
	O	3.05	79.01	-0.350
N_2	N	3.306	38.298	-0.405
	COM	0.00	0.00	0.810
O_2	O	3.04	53.02	-0.112
	COM	0.00	0.00	0.224

Supplementary Table 4: Calculated textural characteristics of ZIF-76.

Pore volume (cm^3/g)	PLD (Å)	LCD (Å)
0.84	3.24	15.91

Supplementary Methods

Simulated Gas Adsorption Isotherms: In GCMC simulations, Lennard-Jones (LJ) 12-6 and Coulomb potentials were used to model repulsion-dispersion forces and electrostatic interactions, respectively. The Lorentz-Berthelot mixing rules were used to calculate adsorbent-adsorbate and adsorbate-adsorbate LJ cross interaction parameters. Molecular simulations were performed for 10,000 cycles with the first 5,000 cycles for initialization and the last 5,000 cycles for taking ensemble averages. Three different types of moves including translation, reinsertion and swap of a molecule were considered for spherical molecules. Rotation move was also applied for non-spherical molecules. The cut-off distance was set to 12.8 Å for truncation of the intermolecular interactions and simulation cell lengths were increased to at least 26 Å along each dimension. Periodic boundary conditions were applied in all simulations. Peng-Robinson equation of state was used to convert the pressure to the corresponding fugacity. All molecular simulations were performed using a rigid framework. More details of these simulations can be found in the literature.[1,2]

Single-site spherical Lennard-Jones (LJ) 12-6 potential was used to model H_2,[3] Ar,[4] and CH_4.[5] CO_2 was modeled as a three site linear molecule with three charged LJ interaction sites located at each atom using the EPM2 potential[6]. Similarly, N_2 (O_2) was modeled as a three site molecule with two sites located at two N (O) atoms and the third one located at its center of mass (COM) with partial point charges and the potential parameters of N_2 and O_2 were taken from the literature.[7] The interaction potential parameters for gas molecules were given in Supplementary Table 3. The atomic charges of ZIF-76 were estimated using charge equilibration method as implemented in RASPA simulation code.[8] The Ewald summation method was used to calculate electrostatic interactions. The potential parameters of ZIF-76 atoms were taken from the Dreiding force field.[9] These potentials and force fields were selected based on the results of previous studies which showed good agreement between simulation results and experimentally measured gas uptake data of ZIFs.[10,11]

In order to compare simulation results with the experimentally measured gas uptake data, the absolute gas amount (N_{abs}) obtained directly from GCMC simulations were converted to the excess gas amount (N_{ex}) as follows:

$$N_{ex} = N_{abs} - \rho_g \cdot V_g \qquad (1)$$

where ρ_g is the density of adsorbates in gas phase obtained from the Peng-Robinson equation of state and V_g is the pore volume of ZIF-76.

RASPA was used to compute gas adsorption isotherms. Zeo++ software[12] was used to compute physical properties of ZIF-76 such as density, pore limiting diameter (PLD), the largest cavity

diameter (LCD) and pore volume. The accessible pore volume is defined as the volume reachable by the center of the probe and calculated using a Monte Carlo integration technique. Pore volume calculations were performed using a probe radius of 0 Å.

The structure of ZIF-76 is highly disordered, where imidazolate (Im) linker positions are partially occupied by chlorobenzimidazolate (cbIm). The disorder was removed manually to give the literature[13] linker ratio of cbIm/Im = 1/3. The geometry was then optimized prior to molecular simulations. Steepest descent minimization was performed using the Forcite module of Materials Studio 8.0.[14] Note: Perez-Pellitero *et al.*[13] examined different possibilities for the position and orientation of the organic linkers using different configurations and they showed that adsorption results were not affected dramatically.

Supplementary References

1 Dubbeldam, D., Torres-Knoop, A. & Walton, K. S. On the Inner Workings of Monte Carlo Codes. *Mol. Simul.* **39**, 1253–1292 (2013).

2 Frenkel, D. & Smit, B. *Understanding Molecular Simulation: From Algorithms to Applications*. 2nd edn, (Academic Press, 2002).

3 Buch, V. Path Integral Simulations of Mixed *Para*-D_2 And *Ortho*-D_2 Clusters: The Orientational Effects. *J. Chem. Phys.* **100**, 7610–7629 (1994).

4 Boato, G. & Casanova, G. A self-consistent set of molecular parameters for neon, argon, krypton and xenon. *Physica* **27**, 571-589 (1961).

5 Martin, M. G. & Siepmann, J. I. Transferable Potentials For Phase Equilibria. 1. United-Atom Description of N-Alkanes. *J. Phys. Chem. B* **102**, 2569–2577 (1998).

6 Potoff, J. J. & Siepmann, J. I. Vapor-Liquid Equilibria of Mixtures Containing Alkanes, Carbon Dioxide, and Nitrogen. *AIChE J.* **47**, 1676-1682 (2001).

7 Calero, S., Martín-Calvo, A., Hamad, S. & García-Pérez, E. On the performance of Cu-BTC metal organic framework for carbon tetrachloride gas removal. *Chem. Commun.* **47**, 508-510 (2011).

8 Dubbeldam, D., Calero, S., Ellis, D. E. & Snurr, R. Q. RASPA: molecular simulation software for adsorption and diffusion in flexible nanoporous materials. *Mol. Sim.* **42**, 81-101 (2016).

9 Mayo, S. L., Olafson, B. D. & Goddard, W. A. DREIDING: a generic force field for molecular simulations. *J. Phys. Chem.* **94**, 8897-8909 (1990).

10 Rankin, R. B., Liu, J., Kulkarni, A. D. & Johnson, J. K. Adsorption and Diffusion of Light Gases in ZIF-68 and ZIF-70: A Simulation Study. *J. Phys. Chem. C* **113**, 16906-16914 (2009).

11 Timón, V., Senent, M. L. & Hochlaf, M. Structural single and multiple molecular adsorption of CO2 and H2O in zeolitic imidazolate framework (ZIF) crystals. *Microporous Mesoporous Mater.* **218**, 33-41 (2015).

12 Willems, T. F., Rycroft, C. H., Kazi, M., Meza, J. C. & Haranczyk, M. Algorithms and Tools for High-Throughput Geometry-Based Analysis of Crystalline Porous Materials. *Microporous Mesoporous Mater.* **149**, 134–141 (2012).

13 Pérez-Pellitero, J. *et al.* Adsorption of CO2, CH4, and N2 on zeolitic imidazolate frameworks: experiments and simulations. *Chem. - Eur. J.* **16**, 1560-1571 (2010).

14 Materials Studio v8.0. (Biovia Software Inc., S.D., CA 92121,USA.).
