# Supplementary material for: Metal-organic framework glasses with permanent accessible porosity
Source: Nat Commun. 2018 Nov 28;9:5042. doi: 10.1038/s41467-018-07532-z (PMC6262007; doi:10.1038/s41467-018-07532-z)
Supplement: Supplementary file 2 — Description of Additional Supplementary Files [file 41467_2018_7532_MOESM2_ESM.pdf]

Description of Additional Supplementary Files

Supplementary Data 1: CIF File of ZIF-76 used for gas sorption simulations
