# Supplementary material for: Metal-organic framework glasses with permanent accessible porosity
Source: Nat Commun. 2018 Nov 28;9:5042. doi: 10.1038/s41467-018-07532-z (PMC6262007; doi:10.1038/s41467-018-07532-z)
Supplement: Supplementary file 3 — Supplementary Data 1 [file 41467_2018_7532_MOESM3_ESM.rtf]

Cif file for ZIF-76 used as input for molecular simulationsdata_zif76edited_symmetry_cell_setting           triclinic_symmetry_space_group_name_H-M   'P 1'_symmetry_Int_Tables_number      1_space_group_name_Hall           'P 1'loop__symmetry_equiv_pos_site_id_symmetry_equiv_pos_as_xyz1 x,y,z_cell_length_a                   22.6702_cell_length_b                   22.6702_cell_length_c                   22.6702_cell_angle_alpha                90.0000_cell_angle_beta                 90.0000_cell_angle_gamma                90.0000_cell_volume                     11651.1loop__atom_site_label_atom_site_type_symbol_atom_site_fract_x_atom_site_fract_y_atom_site_fract_zC1 C 0.00624 0.27237 0.27158H18 H 0.01567 0.23907 0.23874Zn92 Zn 0.00118 0.18339 0.36792Zn96 Zn 0.00068 0.36786 0.18137C6 C 0.15785 0.11609 0.31263C7 C 0.11376 0.15787 0.30965N8 N 0.14062 0.07252 0.34907N9 N 0.06869 0.14037 0.34400C10 C 0.08624 0.08840 0.36712H22 H 0.06036 0.06328 0.39835H23 H 0.19993 0.11768 0.29023H24 H 0.11544 0.19925 0.28600C11 C 0.15750 0.31163 0.11432C12 C 0.11339 0.30905 0.15617N13 N 0.06853 0.34371 0.13869N14 N 0.14047 0.34814 0.07075C15 C 0.08622 0.36659 0.0866H19 H 0.19945 0.28895 0.11584 H20 H 0.11490 0.28535 0.19753H21 H 0.06056 0.39799 0.06154N16 N 0.01092 0.45115 0.18872Zn40 Zn 0.18223 0.00229 0.36786N17 N 0.01173 0.19055 0.45112Zn34 Zn 0.18179 0.36778 0.00059C25 C -0.00001 0.72589 0.72766C30 C 0.01842 0.49797 0.82619C31 C 0.01700 0.82425 0.49919H37 H 0.06306 0.49856 0.84303H38 H 0.06190 0.84037 0.50021H39 H 0.00351 0.76003 0.76080N35 N 0.06630 0.65209 0.86226N36 N 0.06708 0.85706 0.65112C2 C 0.98518 0.35477 0.31055C3 C 0.98550 0.31246 0.35356N4 N 0.99863 0.33049 0.25808N5 N 0.99914 0.25975 0.32997N47 N 0.81284 0.01310 0.55259C53 C 0.66771 0.40979 0.02191C54 C 0.96875 0.32515 0.41146C55 C 0.58208 0.01750 0.32801C56 C 0.58628 0.31617 0.03347C57 C 0.66946 0.02585 0.41599C58 C 0.96812 0.41271 0.32244C59 C 0.88934 0.30903 0.15556C60 C 0.84584 0.11584 0.30832C61 C 0.69032 0.15293 0.11122C62 C 0.69200 0.11025 0.15445C63 C 0.88925 0.15842 0.31021C64 C 0.84615 0.30671 0.11278C65 C 0.79881 0.06785 0.53485C66 C 0.46564 0.06532 0.20259C67 C 0.79021 0.53139 0.06728C68 C 0.79099 0.47106 0.06785C69 C 0.52593 0.06642 0.20274C70 C 0.79953 0.07006 0.47462C71 C 0.56657 0.03311 0.38579C72 C 0.61018 0.03816 0.42954C73 C 0.95201 0.42704 0.38053C74 C 0.95226 0.38346 0.42495C75 C 0.56915 0.37419 0.04661C76 C 0.60929 0.42111 0.03950Cl77 Cl 0.58634 0.49369 0.05341Cl78 Cl 0.59043 0.06237 0.50028Cl79 Cl 0.92989 0.40177 0.49670N80 N 0.86130 0.07156 0.34455N81 N 0.65571 0.06613 0.13869N82 N 0.93180 0.14095 0.34776N83 N 0.93141 0.34732 0.13851N84 N 0.86117 0.34343 0.06874N85 N 0.65283 0.13561 0.06856N86 N 0.80935 0.55053 0.01461N87 N 0.44713 0.01162 0.18579N88 N 0.81208 0.01619 0.45328N89 N 0.80862 0.45121 0.01481N90 N 0.54646 0.01288 0.18766Zn93 Zn 0.81578 0.36799 0.00296Zn94 Zn 0.81633 0.00596 0.36966Zn95 Zn 0.62993 0.00128 0.18358C100 C 0.63288 0.08259 0.08629C101 C 0.91374 0.08790 0.36755C102 C 0.91320 0.36716 0.08549H131 H 0.54792 0.01021 0.29629H132 H 0.55553 0.28063 0.03959H133 H 0.69722 0.44589 0.01417H134 H 0.96699 0.29150 0.44492H135 H 0.88930 0.19991 0.28664H136 H 0.52453 0.38284 0.06131H137 H 0.55351 0.10371 0.21395H138 H 0.78979 0.10429 0.56428H139 H 0.79042 0.10833 0.44763H140 H 0.71607 0.11228 0.19559H141 H 0.96573 0.44576 0.28853H142 H 0.52072 0.04098 0.39665H143 H 0.43670 0.10180 0.21299H144 H 0.80484 0.11799 0.28402H145 H 0.80555 0.28171 0.11461H146 H 0.77798 0.55988 0.10354H147 H 0.88964 0.28514 0.19686H148 H 0.71397 0.19437 0.11133H149 H 0.70160 0.02672 0.45042H150 H 0.77887 0.44299 0.10448H151 H 0.93853 0.47168 0.39089H153 H 0.93647 0.40033 0.06030H154 H 0.60011 0.05734 0.06252H155 H 0.93738 0.06246 0.40026C165 C 0.68461 0.35122 0.01289C166 C 0.64432 0.30645 0.01545N167 N 0.67053 0.25477 0.00327N168 N 0.73895 0.32910 0.00277C326 C 0.72562 0.00060 0.27442C327 C 0.64177 0.01085 0.31450C328 C 0.68422 0.01382 0.35730N329 N 0.73814 0.00672 0.33307N330 N 0.66686 0.00295 0.26128C103 C 0.26919 0.00696 0.26937N107 N 0.25869 0.00120 0.32843C108 C 0.17748 0.01999 0.50100H152 H 0.23492 0.01702 0.23775H162 H 0.16151 0.06495 0.50160Zn369 Zn 0.36411 0.00003 0.17704C109 C 0.27249 0.27193 0.00202C114 C 0.49981 0.17672 0.01827C115 C 0.17644 0.50042 0.02109H159 H 0.16295 0.49989 0.06686H161 H 0.49869 0.16521 0.06456H163 H 0.23928 0.23839 0.00895C41 C 0.72862 0.00405 0.73058C46 C 0.49801 0.01782 0.82082H51 H 0.49781 0.06303 0.83611H52 H 0.76027 0.01379 0.76492Zn256 Zn 0.82051 0.00021 0.63557C121 C 0.30957 0.15688 0.10867C122 C 0.30593 0.11291 0.15060N123 N 0.34037 0.06768 0.13371N124 N 0.34648 0.13942 0.06557C125 C 0.36415 0.08503 0.08193H156 H 0.39572 0.05903 0.05736H157 H 0.28182 0.11483 0.19171H158 H 0.28741 0.19910 0.10996Zn127 Zn 0.17838 0.63354 0.00115Zn252 Zn 0.81873 0.63335 0.00226N49 N 0.65144 0.06767 0.85994N50 N 0.86331 0.06753 0.66033Zn91 Zn 0.63342 0.17803 0.99950C97 C 0.82077 0.50070 0.98348C164 C 0.72839 0.27007 0.99689H270 H 0.76295 0.23816 0.98895H271 H 0.83838 0.50047 0.93913C110 C 0.35501 0.31270 0.98511C111 C 0.31244 0.35535 0.98834N112 N 0.25942 0.33065 0.99860N113 N 0.33090 0.25908 0.99442C169 C 0.41318 0.32685 0.97017C170 C 0.31815 0.03715 0.59135C171 C 0.04457 0.40813 0.68842C172 C 0.32581 0.41472 0.97901C173 C 0.03167 0.32811 0.59378C174 C 0.40660 0.03633 0.67945C175 C 0.30721 0.10763 0.84358C176 C 0.10987 0.15089 0.69504C177 C 0.15225 0.30497 0.89225C178 C 0.10917 0.30677 0.84944C179 C 0.15260 0.10766 0.69256C180 C 0.30532 0.15104 0.88613C181 C 0.19989 0.53159 0.93524C182 C 0.53205 0.19639 0.93189C183 C 0.06611 0.20344 0.47070C184 C 0.47184 0.19517 0.93015C185 C 0.06651 0.20124 0.53092C186 C 0.19910 0.47132 0.93430C187 C 0.05577 0.38436 0.58407C188 C 0.42637 0.38560 0.95531C189 C 0.38293 0.42950 0.96055C190 C 0.41955 0.05655 0.62233C191 C 0.37554 0.05692 0.57834C192 C 0.06388 0.42364 0.63154Cl193 Cl 0.39207 0.08391 0.50774Cl194 Cl 0.10082 0.49063 0.61999Cl195 Cl 0.49618 0.40423 0.92769N196 N 0.06688 0.34485 0.86634N197 N 0.13674 0.34178 0.93602N198 N 0.34180 0.13573 0.93026N199 N 0.13554 0.06578 0.65407N200 N 0.34503 0.06525 0.86092N201 N 0.06584 0.13607 0.65822N202 N 0.18561 0.55045 0.98954N203 N 0.55028 0.18470 0.98702N204 N 0.18600 0.45114 0.98850N205 N 0.01192 0.18880 0.55046N206 N 0.45094 0.18474 0.98464Zn207 Zn 0.36736 0.18086 0.99546Zn208 Zn 0.00033 0.18196 0.63378C210 C 0.08254 0.08412 0.63428C211 C 0.36494 0.08338 0.91387C212 C 0.08464 0.36504 0.91924H281 H 0.06904 0.39703 0.53997H282 H 0.20847 0.44351 0.89668H283 H 0.39356 0.47501 0.95069H284 H 0.46343 0.07261 0.61241H285 H 0.06120 0.39833 0.94411H287 H 0.39791 0.05993 0.93915H289 H 0.05738 0.06105 0.60095H309 H 0.10418 0.20897 0.55918H310 H 0.43985 0.03811 0.71329H311 H 0.28351 0.10746 0.80217H312 H 0.44742 0.29458 0.96885H313 H 0.11175 0.19140 0.72017H314 H 0.29452 0.44922 0.98638H315 H 0.19318 0.28051 0.89050H316 H 0.10918 0.28285 0.80816H317 H 0.05191 0.43755 0.72499H318 H 0.19389 0.10723 0.71646H319 H 0.28092 0.19199 0.88404H320 H 0.20922 0.56028 0.89828H321 H 0.10356 0.21235 0.44253H322 H 0.28408 0.03968 0.55841H323 H 0.56113 0.20421 0.89489H324 H 0.02401 0.29954 0.55699H325 H 0.44455 0.20268 0.89174C424 C 0.30689 0.01622 0.64817C425 C 0.34968 0.01582 0.69068C454 C 0.01683 0.31226 0.65138C455 C 0.01995 0.35223 0.69639N456 N 0.00140 0.25883 0.67262N457 N 0.00272 0.32701 0.74710C261 C 0.68838 0.11087 0.84270C262 C 0.69235 0.15238 0.88701N263 N 0.65812 0.13508 0.93222C264 C 0.63417 0.08350 0.91457H273 H 0.71031 0.11252 0.80038H274 H 0.60265 0.05878 0.94049H277 H 0.71633 0.19359 0.88531C265 C 0.84076 0.31135 0.88985C266 C 0.88509 0.30742 0.84837N267 N 0.93032 0.34158 0.86573N268 N 0.85799 0.34820 0.93311C269 C 0.91262 0.36556 0.91729H278 H 0.93864 0.39686 0.94219H279 H 0.88349 0.28324 0.80728H280 H 0.79850 0.28930 0.88823Zn33 Zn 0.99846 0.36479 0.82327C116 C 0.72909 0.72978 0.01197N119 N 0.67158 0.74347 0.00082N120 N 0.74304 0.67232 0.00077H160 H 0.76113 0.76214 0.02597Zn253 Zn 0.63301 0.81944 0.00346N126 N 0.55009 0.80998 0.01336N129 N 0.65593 0.86261 0.07133N130 N 0.86566 0.65987 0.06606C98 C 0.49740 0.98058 0.17610C99 C 0.82170 0.98256 0.50185H406 H 0.75939 0.99526 0.24115H407 H 0.83509 0.93678 0.50032H408 H 0.49826 0.93543 0.16069C104 C 0.31238 0.98759 0.35008C105 C 0.35212 0.98264 0.30503N106 N 0.32643 0.99701 0.25361C331 C 0.41043 0.66857 0.01989C332 C 0.32870 0.97635 0.40835C333 C 0.03186 0.59503 0.32953C334 C 0.31719 0.58753 0.03672C335 C 0.04394 0.68993 0.40940C336 C 0.40841 0.95927 0.31405C337 C 0.30679 0.88842 0.15094C338 C 0.10845 0.85080 0.30806C339 C 0.15551 0.68997 0.11404C340 C 0.11275 0.69264 0.15723C341 C 0.15154 0.89361 0.30582C342 C 0.30522 0.84522 0.10817C343 C 0.53026 0.79196 0.06616C344 C 0.06507 0.47067 0.20244C345 C 0.20427 0.93435 0.53007C346 C 0.20415 0.93514 0.46979C347 C 0.46996 0.79220 0.06575C348 C 0.06515 0.53089 0.20177C349 C 0.37550 0.57159 0.05089C350 C 0.06309 0.63322 0.42524C351 C 0.42216 0.61135 0.04052C352 C 0.05585 0.58556 0.38595C353 C 0.42444 0.94334 0.37185C354 C 0.38535 0.95368 0.41907Cl355 Cl 0.49178 0.90758 0.38502Cl356 Cl 0.39011 0.50364 0.08486Cl357 Cl 0.07742 0.51440 0.40736N358 N 0.34221 0.86058 0.06448N359 N 0.06758 0.65788 0.14103N360 N 0.13712 0.65344 0.07097N361 N 0.06647 0.86767 0.34652N362 N 0.13645 0.93745 0.34272N363 N 0.34490 0.93075 0.13418N364 N 0.18739 0.98740 0.55023N365 N 0.01058 0.55060 0.18938N366 N 0.18886 0.98925 0.45093N367 N 0.45083 0.80881 0.01213Zn368 Zn 0.00024 0.63446 0.18370Zn369 Zn 0.36411 1.00003 0.17704C373 C 0.08348 0.63503 0.08846C374 C 0.08453 0.92062 0.36652C375 C 0.36537 0.91277 0.08137H385 H 0.28140 0.55761 0.04562H386 H 0.10812 0.80961 0.28397H387 H 0.28059 0.80439 0.10981H388 H 0.10255 0.44248 0.21117H389 H 0.21466 0.89697 0.55784H390 H 0.02455 0.55832 0.30072H391 H 0.55826 0.78074 0.10312H392 H 0.21488 0.89860 0.44104H393 H 0.44139 0.78063 0.10218H394 H 0.19756 0.71250 0.11432H395 H 0.39843 0.94308 0.46377H396 H 0.10268 0.55905 0.21047H397 H 0.46720 0.59838 0.04968H398 H 0.19217 0.89187 0.28086H399 H 0.30007 0.98573 0.44480H400 H 0.43770 0.95022 0.27778H401 H 0.05065 0.72626 0.43914H402 H 0.11520 0.71659 0.19843H403 H 0.44642 0.69784 0.01061H404 H 0.28251 0.88857 0.19202H405 H 0.08267 0.62609 0.46821H415 H 0.06131 0.94563 0.39986H416 H 0.05697 0.60371 0.06409H417 H 0.39858 0.93631 0.05648C419 C 0.35175 0.68473 0.01072C420 C 0.30714 0.64446 0.01539N421 N 0.25520 0.67029 0.00320C468 C 0.01936 0.69767 0.35351C469 C 0.01621 0.65247 0.31376N470 N 0.00154 0.74820 0.32834C25 C 0.99999 0.72589 0.72766C26 C 0.99260 0.64161 0.68788C27 C 0.99091 0.68366 0.64462N28 N 0.99775 0.66723 0.74124N29 N 0.99620 0.73781 0.66868C42 C 0.64804 0.98423 0.68729C43 C 0.69294 0.98153 0.64719N44 N 0.74416 0.99614 0.67300N45 N 0.66976 0.99695 0.74125C117 C 0.69205 0.64985 0.98053C118 C 0.64926 0.69231 0.98071C213 C 0.68191 0.59384 0.95714C214 C 0.98202 0.66836 0.58551C215 C 0.58987 0.97250 0.67071C216 C 0.59309 0.68162 0.95787C217 C 0.68402 0.96093 0.58989C218 C 0.98782 0.58170 0.67444C219 C 0.88537 0.69012 0.84558C220 C 0.84548 0.88832 0.69206C221 C 0.69097 0.84571 0.89203C222 C 0.69283 0.88839 0.84873C223 C 0.88794 0.84486 0.69362C224 C 0.84197 0.68779 0.88817C225 C 0.93254 0.79716 0.46718C226 C 0.46812 0.93228 0.79465C227 C 0.93451 0.46666 0.79662C228 C 0.52838 0.93200 0.79637C229 C 0.93343 0.52693 0.79739C230 C 0.93164 0.79587 0.52746C231 C 0.97512 0.56556 0.61613C232 C 0.57928 0.95231 0.61299C233 C 0.62651 0.94526 0.57318C234 C 0.62540 0.58136 0.93493C235 C 0.97133 0.60879 0.57187C236 C 0.58101 0.62500 0.93546Cl237 Cl 0.51099 0.60900 0.90671Cl238 Cl 0.95073 0.58804 0.50030Cl239 Cl 0.50725 0.93513 0.59055N240 N 0.93202 0.86041 0.65721N241 N 0.86289 0.93092 0.65454N242 N 0.65586 0.93220 0.86377N243 N 0.65271 0.86259 0.93418N244 N 0.93026 0.65527 0.86248N245 N 0.85955 0.65125 0.93154N246 N 0.98766 0.44788 0.81476N247 N 0.98594 0.81507 0.44885N248 N 0.44846 0.98594 0.81015N249 N 0.98506 0.81123 0.54808N250 N 0.54781 0.98603 0.81120N251 N 0.98648 0.54722 0.81446Zn254 Zn 0.99686 0.81551 0.63139Zn255 Zn 0.99719 0.63075 0.81934Zn257 Zn 0.63112 0.99773 0.81812C258 C 0.91339 0.63253 0.91482C259 C 0.63241 0.91542 0.91592C260 C 0.91575 0.91290 0.63442H272 H 0.59916 0.94045 0.93926H275 H 0.94107 0.93659 0.60164H276 H 0.93940 0.60124 0.93974H286 H 0.71615 0.56101 0.95481H288 H 0.98251 0.70034 0.55082H290 H 0.55346 0.98001 0.69987H291 H 0.71518 0.80459 0.89235H292 H 0.55994 0.71557 0.95614H293 H 0.80019 0.71083 0.88745H294 H 0.89834 0.43790 0.78475H295 H 0.99447 0.54783 0.70658H296 H 0.80413 0.88843 0.71587H297 H 0.43999 0.89517 0.78429H298 H 0.61846 0.92863 0.52914H299 H 0.88582 0.80376 0.71775H300 H 0.71774 0.88666 0.80807H301 H 0.55673 0.89484 0.78677H302 H 0.88350 0.71389 0.80426H303 H 0.89652 0.55468 0.78538H304 H 0.89454 0.78385 0.55495H305 H 0.96856 0.51952 0.60525H306 H 0.61624 0.53814 0.91681H307 H 0.89614 0.78676 0.43815H308 H 0.71992 0.95471 0.55953C376 C 0.68689 0.88801 0.16038C377 C 0.69026 0.84580 0.11670N378 N 0.65022 0.93114 0.14259C379 C 0.63241 0.91458 0.08833H410 H 0.70913 0.88702 0.20257H411 H 0.60111 0.93925 0.06211H414 H 0.71425 0.80465 0.11887C380 C 0.88453 0.85113 0.30763C381 C 0.84060 0.89311 0.31131N382 N 0.92963 0.86776 0.34235N383 N 0.85796 0.93600 0.34852C384 C 0.91226 0.91945 0.36632H409 H 0.93819 0.94384 0.39808H412 H 0.88269 0.81018 0.28322H413 H 0.79848 0.89202 0.28895Zn32 Zn 0.99746 0.82460 0.36596Zn48 Zn 0.36526 0.99609 0.81833Zn128 Zn 0.17861 0.99670 0.63339Zn370 Zn 0.36783 0.81531 0.99936C372 C 0.50070 0.82070 0.98131C418 C 0.27028 0.72792 0.99485N422 N 0.32933 0.73865 0.99957H441 H 0.23818 0.76227 0.98675H452 H 0.50105 0.83761 0.93668C423 C 0.26868 0.98914 0.72919N426 N 0.32666 0.99832 0.74243N427 N 0.25504 0.99898 0.67142H451 H 0.23614 0.97707 0.76191C433 C 0.15088 0.69321 0.88933C434 C 0.10899 0.68971 0.84533N435 N 0.13438 0.65799 0.93410C436 C 0.08280 0.63394 0.91646H445 H 0.11006 0.71234 0.80334H448 H 0.19179 0.71768 0.88767H449 H 0.05868 0.60160 0.94194C428 C 0.31210 0.83903 0.88593C429 C 0.30840 0.88299 0.84410N430 N 0.34226 0.92848 0.86141N431 N 0.34852 0.85678 0.92932C432 C 0.36586 0.91132 0.91328H443 H 0.29022 0.79667 0.88450H444 H 0.28462 0.88093 0.80281H447 H 0.39687 0.93762 0.93823C437 C 0.11053 0.83949 0.68754C438 C 0.15264 0.88330 0.69056N439 N 0.13553 0.92856 0.65625C440 C 0.08343 0.91136 0.63310H442 H 0.05848 0.93747 0.60192H446 H 0.11179 0.79736 0.70987H450 H 0.19391 0.88134 0.71439C209 C 0.97957 0.18095 0.50071C453 C 0.99302 0.26977 0.73127H465 H 0.93372 0.16771 0.50057H466 H 0.98140 0.23579 0.76266C458 C 0.84579 0.11261 0.69462C459 C 0.88713 0.15708 0.69083N460 N 0.93045 0.14007 0.65394C461 C 0.91484 0.08543 0.63648H462 H 0.93969 0.05967 0.60494H463 H 0.80453 0.11415 0.71851H464 H 0.88525 0.19935 0.71283C371 C 0.97854 0.50083 0.18018C467 C 0.99122 0.73204 0.27136N471 N 0.99979 0.67336 0.26051H476 H 0.97951 0.76343 0.23755H480 H 0.93267 0.50065 0.16712C472 C 0.87850 0.71562 0.08127C473 C 0.92065 0.71455 0.12490N474 N 0.93446 0.65814 0.13732C475 C 0.90034 0.62599 0.10078H477 H 0.94280 0.75254 0.14317H478 H 0.86111 0.75440 0.05968H479 H 0.90410 0.57871 0.09616#END
